# New Cathode Materials in the Fe‐PO_4_‐F Chemical Space for High‐Performance Sodium‐Ion Storage

**DOI:** 10.1002/advs.202200924

**Published:** 2022-05-26

**Authors:** Xuelian Liu, Jiande Wang, Mengyuan Du, Koen Robeyns, Yaroslav Filinchuk, Qi Zhu, Varun Kumar, Yann Garcia, Gheorghe Borodi, Cristian Morari, Jean‐Francois Gohy, Alexandru Vlad

**Affiliations:** ^1^ Institute of Condensed Matter and Nanosciences Université catholique de Louvain Louvain‐la‐Neuve B‐1348 Belgium; ^2^ Institutul National de Cercetare‐Dezvoltare pentru Tehnologii Izotopice si Moleculare Cluj‐Napoca Str. Donat nr. 67‐103, PO 5 Box 700 Cluj‐Napoca 400293 Romania

**Keywords:** cathode material, phosphate fluoride materials, polyanionic framework, sodium‐ion battery

## Abstract

Sodium and iron make up the perfect combination for the growing demand for sustainable energy storage systems, given the natural abundance and sustainability of the two building block elements. However, most sodium–iron electrode chemistries are plagued by intrinsic low energy densities with continuous ongoing efforts to solve this. Herein, the chemical space of a series of (meta)stable, off‐stoichiometric Fe‐PO_4_‐F phases is analyzed. Some are found to display markedly improved electrochemical activity for sodium storage, as compared to the amorphous or thermodynamically stable phases of equivalent composition. The metastable crystalline Na_1.2_Fe_1.2_PO_4_F_0.6_ delivers a reversible capacity of more than 140 mAh g^−1^ with an average discharge potential of 2.9 V (vs Na^+^/Na^0^) resulting in a practical specific energy density of 400 Wh kg^−1^ (estimated at the material level), outperforming many developed Fe‐PO_4_ analogs thus far, with further multiple possibilities to be explored toward improved energy storage metrics. Overall, this study unlocks the possibilities of off‐stoichiometric Fe‐PO_4_‐F cathode materials and reveals the importance to explore the oft‐overlooked metastable or transient state materials for energy storage.

## Graphical abstract

A series of new cathode materials are developed with combinations of not only the advantages in Na‐Fe‐PO_4_‐F building blocks but also compositional diversity and phase activity in this system to attain low‐cost, sustainable, and high‐performance sodium storage materials. The metastable Na_1.2_Fe_1.2_PO_4_F_0.6_ material delivers an energy density of nearly 400 Wh kg^−1^ at the material level, holding promises for Na‐ion batteries.

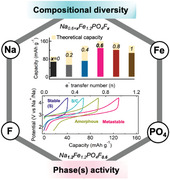

## 1 Introduction

Energy storage plays an important role in our society, from electronic gadgets for daily life to electric vehicles and large‐scale energy storage systems for renewable energy such as solar and wind powder.^[^ ^1^ ^]^ Lithium‐ion batteries (LIBs), as one of the most advanced energy storage systems, have been witnessing great commercial success due to their high energy density and long cycle life, and the demand for LIBs with improved performance continues to grow.^[^ ^2^ ^]^ However, natural resource scarcity and large demand for Li brought recent concerns, leading researchers to look for alternative‐Li chemistries.^[^ ^3^ ^]^ One of the most appealing alternatives is based on sodium storage, especially for large‐scale applications, due to the larger abundance and lower cost of Na sources, and potentially cheaper manufacture.^[^ ^3^, ^4^ ^]^ Sodium‐ion batteries (SIBs) share a similar storage mechanism with established LIBs, but the challenges faced are that the accommodation of Na^+^ in conventional electrode materials is typically more difficult given its larger ionic radius (as compared to that of Li^+^), higher mass, and lower redox potential, resulting overall in lower energy density.^[^ ^5^ ^]^ It has become thus essential to explore and develop electrode materials for SIBs, especially positive electrode chemistries, delivering increased capacity at a high redox potential, while also relying on abundant and sustainable chemistries.

Polyanionic frameworks with ${\mathrm{SO}}_{4}^{2-}$, ${\mathrm{PO}}_{4}^{3-}$, ${\mathrm{BO}}_{3}^{3-}$, and ${\mathrm{SiO}}_{4}^{4-}$ form a major stream of positive electrode material candidates for LIBs and SIBs.^[^ ^6^ ^]^ Compared to layered or disordered oxides, these have a higher redox potential, owing to the inductive effect, as well as stable framework structure which ensures longer‐term cycling stability. Additionally, the reduced safety concerns benefit also from their higher thermal stability.^[^ ^6b^ ^]^ Further energy density increase of these can be attained through substitution with lightweight and electronegative groups like F, N, or OH.^[^ ^6a,d^ ^]^ Fluorinated class of polyanionic cathode materials has thus emerged as a promising chemistry, wherein the higher electronegativity of F translates into enhanced ionic character of the bonds and correspondingly higher redox potential.^[^ ^7^ ^]^

Out of the respective building blocks—that is, Na^+^, transition metal (TM), F^−^, and ${\mathrm{PO}}_{4}^{3-}$—many stoichiometric compositions with promising electrochemical performances have been designed and tested thus far. These can be grouped into the following main classes: NaMPO_4_, NaMPO_4_F, and Na_2_MPO_4_F (where M can be Fe, Mn, Co, V, Cr, or Ni).^[^ ^8^ ^]^ With the polymorphism in these multiplying the possibilities here, off‐stoichiometric compositions further enrich this landscape and have attracted recent attention. For instance, off‐stoichiometric phosphate and sulfate compositions have been found to show better active material utilization at higher redox potentials.^[^ ^9^ ^]^ The off‐stoichiometric chemistries also allow for fine‐tuning of the composition and balancing the Na^+^ content to the equivalent of redox‐active transition metal, while allowing to lower the weight of the anion framework e.g., substituting ${\mathrm{PO}}_{4}^{3-}$ by F^−^). With this respect, Yamada et al. recently reported on a peculiar Fe‐rich Na_0.6_Fe_1.2_PO_4_ phase, which further broadened the off‐stoichiometric positive electrode materials family.^[^ ^10^ ^]^ The Na_0.6_Fe_1.2_PO_4_ phase can be regarded as an Fe‐rich phase (0.4 Na^+^ being replaced with 0.2 Fe^2+^) with potentially 1.2 redox electrons being available, while practically being limited to only 0.6 Na^+^ contained. Fluorine doping, bringing along higher Na^+^ content, could thus be regarded as a means to further increase the specific capacity of this material.

Finally, of essential relevance to developments in this work, it is important to recall that most of today's positive electrode chemistries (e.g., Li[Co, Ni, Mn,…]O_2_, LiFePO_4_, Na_3_V_2_(PO_4_)_3_, and Na_2_FePO_4_F) are typically investigated in their thermodynamically stable form.^[^ ^11^ ^]^ On the contrary, metastable phases are kinetically trapped phases with positive free energy above the equilibrium state, with many of these known to exhibit superior properties than their corresponding stable phases.^[^ ^5^, ^12^ ^]^ Although with moderate interest and applicability so far, these have also started gaining interest in the battery materials field.^[^ ^5^, ^13^ ^]^ Tatsumisago et al. have recently summarized the state‐of‐the‐art progress on glassy and metastable crystalline materials as solid electrolytes and electrode materials, aiming at emphasizing the attention on the underexplored potential of metastable materials for battery applications.^[^ ^14^ ^]^ The metastable phases tend to have larger molar volumes (or less compact) and higher symmetry crystalline structures, favorable thus for ion conduction, and have been proposed for investigation as solid electrolytes.^[^ ^14^ ^]^ Nonetheless, the research and application of this type of materials as cathodes for electrochemical energy storage remains rather limited while the possibilities for metastable phases might be endless, and so the electrochemical properties.

Herein, we explore the above three concepts within one class of battery materials, in which a set of chemistries based only on Na^+^, Fe^2+^, ${\mathrm{PO}}_{4}^{3-}$, and F^−^ are found to be library‐level rich by tuning the stoichiometry of these building blocks while also arresting the systems outside the thermodynamic stability range. Inspired by the knowledge on reported archetypal Na_1 +_ *_x_* FePO_4_F*_x_* (with *x* = 0 or 1) and NaFeF_3_ chemistries, as well as the recently reported Na_0.6_Fe_1.2_PO_4_, we developed a series of linear combinations of these with the empirical formula of Na_0.6 +_ *_x_* Fe_1.2_PO_4_F*_x_* (with analyzed values of *x* = 0, 0.2, 0.4, 0.6, 0.8, and 1).^[^ ^10^, ^15^ ^]^ Likewise the compositional building units (the Na_0.6 +_ *_x_* Fe_1.2_PO_4_F*_x_* could be regarded as a mixture of NaFePO_4_ and NaFeF_3_), we found the thermodynamically stable phases to be electrochemically less active and focused our interest on the transient synthesis states. These are suggested to exist as mixed‐phase compositions.

Altogether combined, this work uncovers the electrochemistry of five crystalline phases based on the diverse Na‐Fe‐PO_4_‐F composition map (by changing *x* in Na_0.6 +_ *_x_* Fe_1.2_PO_4_F*_x_*); with as many as six electrochemically active phases for solely the Na_1.2_Fe_1.2_PO_4_F_0.6_ composition. Through electrochemical and physico‐chemical analyses, we suggest some rationales behind the enhanced electrochemical properties being attributed to optimized composition (equal content of Na^+^ as charge carrier and Fe as redox center and lower content of anions) and metastability of the materials. We also postulate that other low‐energy metastable and transient chemical compositions exist, and can be synthesized for this class, while other compositions based on other than Fe redox center or ${\mathrm{PO}}_{4}^{3-}$ anion framework could be subsequently developed based on the approaches developed in this work.

## 2 Results and Discussion

### 2.1 Synthesis Strategy of the Metastable Materials in the Na_0.6 +_ *_x_* Fe_1.2_PO_4_F*_x_* Chemical Space

**Figure 1:**
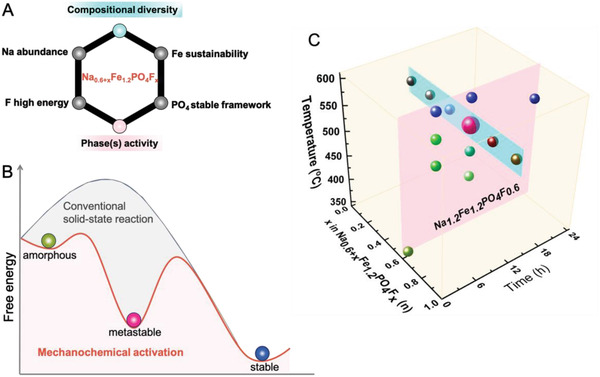
Refinement of design principles. A) Advantages of the Na_0.6 +_ *_x_* Fe_1.2_PO_4_F*_x_* family materials. B) Evolution of a system from an amorphous, to a metastable, finally to a state of stable equilibrium during synthesis (bullets color code corresponds to the samples in the pink cross‐section plane depicted in the phase diagram of panel (C)). C) Phases and composition map of explored combinations in this work. The various composition samples with the change of *x* are within the blue plane, and the various phases for *x* = 0.6 are contained within the pink plane.

Relying on the introductory rationales while focusing on the exotic Fe‐rich, Na‐deficient chemistry proposed by Yamada (i.e., Na_0.6_Fe_1.2_PO_4_), we have undergone this study on mixing the six key essential parameters of high‐performance positive electrode chemistry for the SIBs (**Figure** **1**A). The nominal composition of our study, namely the Na_0.6 +_ *_x_* Fe_1.2_PO_4_F*_x_* synergistically mixes these concepts. The inclusion of fluorine in the parent Na_0.6_Fe_1.2_PO_4_ composition was designed with a twofold interest. First, this increases the Na^+^ content with a minimal penalty on the specific capacity; while second, considering the redox potential increase associated with fluorine doping. Preliminary analysis of synthesis conditions also revealed a series of crystalline intermediates with superior electrochemical properties and the broad plethora of materials found have been thus analyzed. These include: i) the thermodynamically stable phase; ii) the amorphous (or non‐crystalline) phase made by low temperature; as well as the iii) intermediate metastable crystalline mixed‐phase materials (Figure 1B,C).

The Na_0.6 +_ *_x_* Fe_1.2_PO_4_F*_x_* series was obtained by changing the stoichiometry of precursors and annealing under similar conditions. The amorphous, crystalline metastable, and crystalline stable compositions of Na_1.2_Fe_1.2_PO_4_F_0.6_ were obtained by changing the annealing conditions (time or temperature), which can be explained by transition states in the free energy diagram (Figure 1B). Note that other possibilities in the composition—synthesis conditions diagram may exist and remain unexplored. The synthesis design relied on a detailed analysis of the intermediate products in the precursor preparation and annealing processes. The reactants consisted in mixing the desired molar ratios of iron oxalate dihydrate (FeC_2_O_4_•2H_2_O), sodium carbonate (Na_2_CO_3_), ammonium dihydrophosphate (NH_4_H_2_PO_4_), and sodium fluoride (NaF). These were selected so that they can react and decompose at low temperatures with all volatile or gaseous products generation.

**Figure 2:**
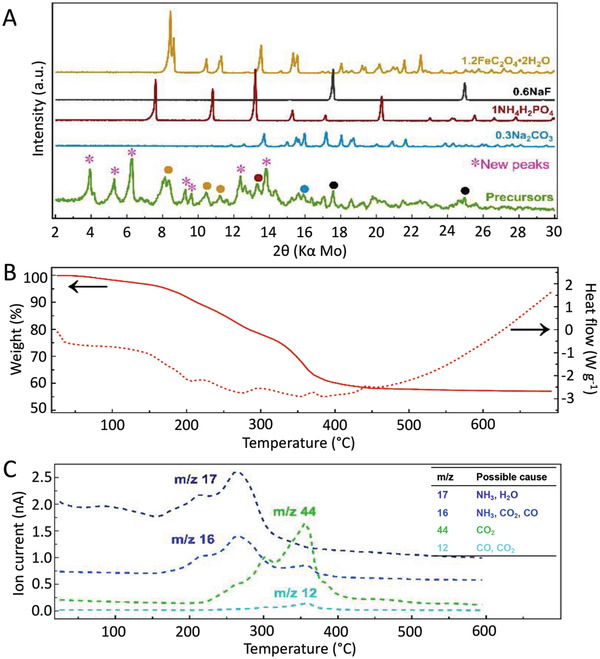
Insight into synthesis process of Na_1.2_Fe_1.2_PO_4_F_0.6_ phases. A) Powder X‐ray diffraction patterns of starting materials and precursors prepared by high‐energy ball milling. B) TGA‐DSC and C) associated MS analysis for the precursors annealing step.

High‐energy ball milling of the reactants was already found to induce major chemical and phase changes (**Figure** **2**A) as a direct consequence of combined mechanochemical and acid–base reactions in the system. It should be noted that the reactants' milling conditions were found to significantly affect the precursors' composition with further impact on the final products (e.g., manual grinding or low‐energy planetary ball milling leading to different phases, Figure S1, Supporting Information). Under high‐energy ball milling conditions, new phases are formed, with also residuals of initial reactants (Figure 2A). The new phases can be roughly indexed to sodium amide phosphate hydrate, sodium imide phosphate hydrate, and/or sodium phosphate hydrate, indicating that not only intimate mixing but also chemical reactions occurred during this step. This can lower the diffusion length and barriers to diffusion for subsequent solid‐state annealing step, which is often favorable to the production of metastable phases.^[^ ^16^, ^17^ ^]^

Combined thermogravimetric analysis, differential scanning calorimetry, and mass spectrometry (TGA‐DSC‐MS) further revealed a series of important features upon which we relied for the synthesis design. The TGA data shows that the major mass losses occur between 100 and 350 °C assigned to the decomposition of the precursors, corresponding to an exothermic process over the same temperature as detected by DSC. The MS survey indicates the removal of H_2_O, NH_3_, CO_2_, and CO as the decomposition products, with no traces of HF, and thus no F loss. The estimated total mass removal at 400 °C corresponds to a remaining composition of Na_0.6 +_ *_x_* Fe_1.2_PO_4_F*_x_* as targeted (this elemental composition is also confirmed by other techniques, Figure S2 and Table S1, Supporting Information). Past this temperature, no further chemical composition change can be detected, and the endothermic processes taking place can be associated with solid‐state diffusion and crystallization processes.

### 2.2 The Chemical Space of Na_0.6 +_ *_x_* Fe_1.2_PO_4_F*_x_* Compositions and Phases

**Figure 3:**
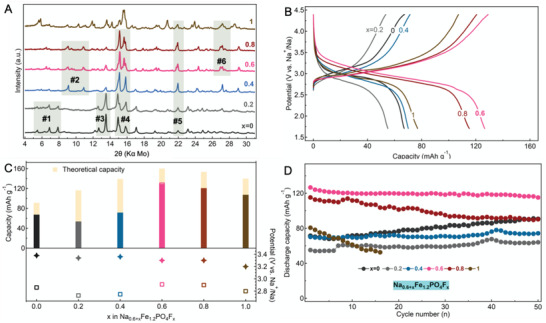
Compositional diversity. A) PXRD survey of Na_0.6 +_ *_x_* Fe_1.2_PO_4_F*_x_* series for various *x* compositions. The bars in grey are eye guidelines to show similarities for specific diffraction regions. B) First cycle potential versus capacity galvanostatic plots for studied compositions. C) Charge capacity (top) and average discharge potential evolution function of *x* in Na_0.6 +_ *_x_* Fe_1.2_PO_4_F*_x_*. The potentials were calculated by dividing the energy density (mAhV g^−1^) by the maximum capacity (mAh g^−1^). D) Cycling stability of the Na_0.6 +_ *_x_* Fe_1.2_PO_4_F*_x_* materials at a current density of 5 mA g^−1^.

**Figure 4:**
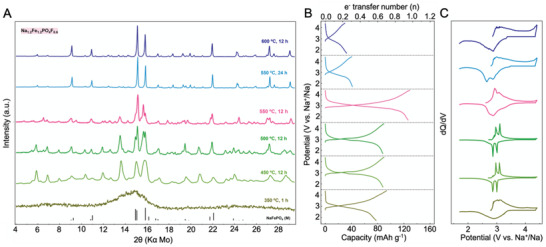
Phase diversity. A) PXRD survey, B) first cycle potential versus capacity profile, and C) corresponding normalized d*Q*/d*V* of the Na_1.2_Fe_1.2_PO_4_F_0.6_ materials.

Based on the three accessible degrees of freedom—annealing temperature, reaction time, and composition (Figure 1C)—we prepared and analyzed 14 pure and mixed phases (Figure 1C). An overview of these is presented in **Figures** **3** and **4**. From these free parameter analyses, the Na_1.2_Fe_1.2_PO_4_F_0.6_ (*x* = 0.6) composition prepared at 550 °C for 12 h was found to display the best performance and was thus selected as the main investigation target, although others were also found to be electrochemically active.

Figure 3 summarizes the analysis of the compositional changes in the Na_0.6 +_ *_x_* Fe_1.2_PO_4_F*_x_* series (*x* varied from 0 to 1 in steps of 0.2). All phases were prepared by annealing for 12 h at 550 °C, which was found to be optimal for the best electrochemically performing Na_1.2_Fe_1.2_PO_4_F_0.6_ composition (refer to Figure 4 for details). The comparative analysis of the powder X‐ray diffraction (PXRD) patterns (Figure 3A) and associated electrochemical properties (Figure 3B–D) reveals a series of peculiar features. The Na_0.6_Fe_1.2_PO_4_ (*x* = 0) phase made in this work shows similarity to the one reported by Yamada et al., indicating the good reproducibility of the method.^[^ ^10^ ^]^ As the *x* increases, the PXRD pattern gradually evolves, while also maintaining similarities for specific diffraction regions (marked with #1–6 in Figure 3A). The two groups of materials (*x* = 0–0.2 and *x* = 0.4–0.8) share similar PXRD patterns separately, attributed to possible mixed phases existing in the respective *x* ranges, as discussed in the following. Little changes in phase structure are noted for low fluorine content, resulting in the materials (*x* = 0 and 0.2) with similar X‐ray diffraction features. Different phases are formed in the middle F‐content range. The broadening and appearance of additional peaks for *x* = 1 imply further new phase formation or separation in progress, and thus sintering at higher temperatures or for a longer time is needed to form a well‐crystallized final product in future investigation.

According to the molar ratio of Na to Fe in the Na_0.6 +_ *_x_* Fe_1.2_PO_4_F*_x_* series, the highest capacity is expected to be attained for *x* = 0.6 with also possibly the redox potential increase proportional to *x*. Both capacity and redox potential follow this rule (Figure 3B,C) with the highest reversible capacity of 127 mAh g^−1^ attained for Na_1.2_Fe_1.2_PO_4_F_0.6_ (at a current density of 5 mA g^−1^ corresponding to a C/32 rate). Interesting to note the analogy and differences between similar groups of materials with, for example, low capacities and low redox potential attained for *x* = 0 and 0.2; as well as for *x* = 1. The phases obtained for *x* = 0.6 and 0.8 follow more closely the theoretical charge storage capacity estimates. The storage capacity difference between Na_1_Fe_1.2_PO_4_F_0.4_ (*x* = 0.4) and Na_1.4_Fe_1.2_PO_4_F_0.8_ (*x* = 0.8) can be explained by Na and Fe deficiency in the former and latter, respectively, while acquiring a higher molecular weight (192.5 vs 209.4 g mol^−1^). The redox potential was also generally found to correlate with fluorine content where F‐rich phases display higher average discharge potential. Stable capacity retention with cycling is observed, with the exception of the end member *x* = 1 (Figure 3D).

Since in the series of Na_0.6 +_ *_x_* Fe_1.2_PO_4_F*_x_*, the composition with *x* = 0.6 displays the best electrochemical performance (Figure 3B), its formation dynamics was next investigated. Starting from the same precursors and processing conditions (Figure 2), different annealing conditions were applied: from the lowest temperature and shortest time (350 °C, 1 h) to gradually the highest temperature and longer annealing conditions (600 °C, 12 h). The PXRD pattern evolution, the first cycle charge–discharge potential profiles, and the corresponding d*Q*/d*V* curves are displayed in Figure 4. The mass loss for the phase prepared at 350 °C for 1 h was measured to be very close to the value from the TGA analysis (Figure 2), indicating a complete decomposition of the starting materials. Under these synthesis conditions, only a broad diffraction pattern can be seen, indicating an amorphous (or non‐crystalline) state, as a result of a process sufficient for precursors decomposition yet not enough for structural ordering—in other words, an intermediate phase in the formation of the thermodynamically stable Na_1.2_Fe_1.2_PO_4_F_0.6_ product (Figure 1B). Surprisingly, this amorphous phase was found to be electrochemically active and deliver ≈80 mAh g^−1^ (corresponding to 0.6 Na^+^ exchange), with sloping potential profiles and a corresponding broad d*Q*/d*V* redox peak.

The material prepared at 500 °C for 12 h displays clear signs of crystallinity (Figure 4A), potentially being composed of more than one phase. The two charge–discharge plateaus centered at 3 V (vs Na^+^/Na) or the corresponding pair of sharp redox peaks in the d*Q*/d*V* plot (Figure 4B,C) could be another indication of a multiple phase composition although additional studies are required to ascertain this. As the annealing temperature is increased to 550 °C, additional diffraction peaks disappear or become weaker (most significant being at 2*Θ* = 5.4°, 7.9°, 10.3°, 11.9°, 13.5°, 14.9°, and 20.8°), with only minor changes detected for annealing durations of 6 and 12 h (both at 550 °C, the main difference being the change in the relative intensities of certain peaks). We assign this to the formation of new phase(s). The two materials share similar galvanostatic potential and d*Q*/d*V* profiles; however, the phase annealed for 12 h displayed considerably higher material electrochemical utilization. An additional 12 h of annealing at 550 °C or processing at higher temperatures (e.g., 600 °C) yielded the thermodynamically stable phase of Na_1.2_Fe_1.2_PO_4_F_0.6_. Surprisingly, the electrochemistry of the end‐series samples displayed the lowest electrochemical activity, with less than 0.3 Na^+^ equivalents being possible to extract (at a current density of 5 mA g^−1^) accompanied also by high polarization.

From this compositional and phase library mapping, the Na_1.2_Fe_1.2_PO_4_F_0.6_ composition clearly stands out as the best performing at this stage with the highest redox potential and electrochemical utilization. Since the amorphous, metastable, as well as stable materials, could be isolated, we characterized these to correlate the phase(s) and structure with the electrochemical activity. For the sake of clarity, hereafter the amorphous phase (annealed for 1 h at 350 °C) is denoted as Na_1.2_Fe_1.2_PO_4_F_0.6__AM (or simplified as _AM); the metastable phase prepared at 550 °C for 12 h as Na_1.2_Fe_1.2_PO_4_F_0.6__M (or simplified as _M); whereas the stable phase (prepared at 550 °C for 24 h or the one at 600 °C for 12 h) is termed as Na_1.2_Fe_1.2_PO_4_F_0.6__S (or simplified as _S). The three will be of central interest in the following analysis.

### 2.3 Comparative Analysis of Na_1.2_Fe_1.2_PO_4_F_0.6__S, _M, and _AM

**Figure 5:**
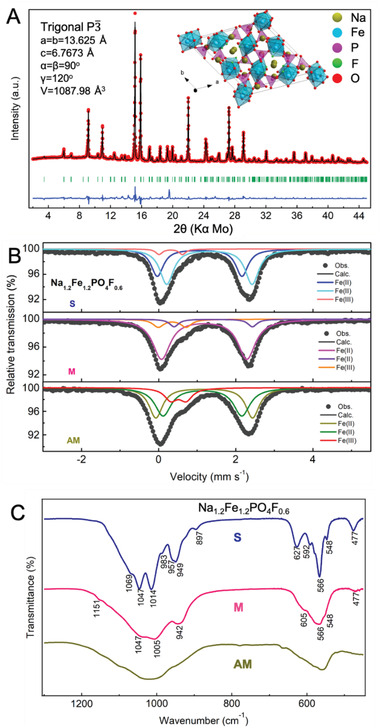
Characterizations of Na_1.2_Fe_1.2_PO_4_F_0.6__S, _M, and _AM. A) PXRD patterns and results of the Rietveld refinement of Na_1.2_Fe_1.2_PO_4_F_0.6__S: the red dots are the measured data, the black line is the calculated pattern, and the blue line is the difference between the calculated and observed data; whereas, the green bars correspond to the expected positions of the Bragg reflection peaks. The inset in (A) shows the crystal structure of Na_1.2_Fe_1.2_PO_4_F_0.6__S along the *c*‐axis and views along other directions are shown in Figure S3, Supporting Information. B) Room‐temperature Mössbauer and C) Fourier transform infrared spectra of Na_1.2_Fe_1.2_PO_4_F_0.6__S, _M, and _AM phases.

The large discrepancy in the crystallinity as well as the electrochemical activity of the two _S (thermodynamically stable) and _M (metastable) phases, while being accessed in a narrow synthesis conditions range, motivated us for additional physico‐chemical analysis. The properties of the _AM (amorphous) phase have also been evaluated alongside for comparison. Indexing of the Na_1.2_Fe_1.2_PO_4_F_0.6__S phase (**Figure** **5**A) revealed its similarity to a reported iron‐based compound Ca_0.1667_Na_3.6667_FeP_2_O_8_F (ICSD #079632)^[^ ^18^ ^]^ with, however, slight variations in the lattice parameters applied for Na_1.2_Fe_1.2_PO_4_F_0.6__S. This was indexed to trigonal P$\bar{3}$ space group, with lattice parameters: *a* = *b* = 13.62512 Å, *c* = 6.76738 Å, *α* = *β* = 90°, *γ* = 120°, and *V* = 1088.007 Å^3^ (Figure 5A and Figure S3 and Table S2, Supporting Information). The structure reveals a 3D framework with corner‐sharing PO_4_ tetrahedral and FeO_6_/FeO_5_F octahedral units. Na^+^ is either located in the tunnels surrounded by the Fe octahedral and P tetrahedral groups or alternated with PO_4_ tetrahedrons along the *c*‐axis. The PXRD indexing of the Na_1.2_Fe_1.2_PO_4_F_0.6__M has been also attempted, but a plausible refinement solution could not be accessed. Although a solution was obtained from synchrotron XRD data, the large unit cell volume of 8297 Å^3^ attained indicates a mixture rather than a single‐phase material for Na_1.2_Fe_1.2_PO_4_F_0.6__M (Figure S4, Supporting Information), requiring further advanced investigation.

The local coordination environment of Fe ions in Na_1.2_Fe_1.2_PO_4_F_0.6__S, _M, and _AM was also investigated with Mössbauer spectroscopy (Figure 5B, the fitting parameters are listed in Table S3, Supporting Information). The spectrum of Na_1.2_Fe_1.2_PO_4_F_0.6__S can be fitted to three doublets, corresponding to two types of Fe(II) environment (accounting for 34% and 61%, respectively) and one type of Fe(III) environment (5%). The relatively high isomer shift (*δ*) values (>1 mm s^−1^) of the two Fe(II) species indicate octahedral coordination (*O*_h_), which agrees well with the FeO_6_ and FeO_5_F octahedra in the crystal structure of the Na_1.2_Fe_1.2_PO_4_F_0.6__S. Only doublets are found, implying no iron oxide formation, that are associated with the sextet signal. The Fe(III) component with low *δ* and ∆*E*_Q_ values present can be due to partial oxidation of Na_1.2_Fe_1.2_PO_4_F_0.6__S during the long acquisition time used (≈2 weeks).

Two Fe(II) (*O*_h_) sites were also detected for Na_1.2_Fe_1.2_PO_4_F_0.6__M, but the corresponding fit parameters are different, implying no presence of the Na_1.2_Fe_1.2_PO_4_F_0.6__S phase in the Na_1.2_Fe_1.2_PO_4_F_0.6__M. The main Fe(II) (*O*_h_) site (accounting for as much as 82%) has *δ* (1.17 mm s^−1^) and a quadrupole splitting (∆*E*_Q_, 2.21 mm s^−1^) parameters close to those of reported Na_2_Fe(II)PO_4_F phase (1.23 and 2.19 mm s^−1^, respectively).^[^ ^19^ ^]^ Note that *δ* = 0.34 and ∆*E*_Q_ = 0.72 mm s^−1^ of Fe(III) (*O*_h_) for Na_1.2_Fe_1.2_PO_4_F_0.6__M are roughly equivalent to those for NaFe(III)PO_4_F (*δ* = 0.37 and ∆*E*_Q_ = 0.71 mm s^−1^), together with the similar parameters of the Fe(II) site mentioned above, implying that Na_1.2_Fe_1.2_PO_4_F_0.6__M possesses similar Fe environment to that in the Na_2_Fe(II)PO_4_F and the minor Fe(III) species originate from the air‐exposure oxidation of the main Fe(II) site component rather than the weak one.^[^ ^20^ ^]^

The Na_1.2_Fe_1.2_PO_4_F_0.6__AM has two Fe(II) (*O*_h_) sites with different *δ* and ∆*E*_Q_ values from the Na_1.2_Fe_1.2_PO_4_F_0.6__S and_M, but the values are found to be very close to those of Na_0.6_Fe_1.2_PO_4_ (refer to Table S3, Supporting Information), indicating the similar Fe environment in Na_1.2_Fe_1.2_PO_4_F_0.6__AM and Na_0.6_Fe_1.2_PO_4_. This could imply the existence of Na_0.6_Fe_1.2_PO_4_ or a similar compound in an amorphous state in the Na_1.2_Fe_1.2_PO_4_F_0.6__AM. Together with further assessment and comparison of PXRD pattern evolutions in Figures 3 and 4, where some peaks, especially at 13.5° and 14.9° found as two main peaks in the pattern of Na_0.6_Fe_1.2_PO_4_ (*x* = 0) become less pronounced either as *x* (the content of NaF) increases or annealing temperature elevates for Na_1.2_Fe_1.2_PO_4_F_0.6_ composition, it can be assumed that Na_0.6_Fe_1.2_PO_4_ may tend to form first and then get converted to Na_0.6 +_ *_x_* Fe_1.2_PO_4_F*_x_* material(s) upon further annealing.

The vibrational modes of PO_4_^3−^ units of Na_1.2_Fe_1.2_PO_4_F_0.6__S, _M, and _AM materials were also analyzed by Fourier transform infrared (FT‐IR) spectroscopy (Figure 5C). The obtained spectra are consistent with those for maricite NaFePO_4_, Na_2_FePO_4_F, and Li_2_CoPO_4_F, with symmetric *ν*_1_ and asymmetric *ν*_3_ stretching modes located in the high‐wavenumber region (900–1200 cm^−1^), and the symmetric *ν*_2_ and asymmetric *ν*_4_ bending modes in the 450 to 650 cm^−1^ range.^[^ ^21^ ^]^ The Na_1.2_Fe_1.2_PO_4_F_0.6__S, _M, and _AM share similar bands, despite minor shifts. The broadening of the vibration bands is without exception identified in the case of _M and _AM materials as compared to the stable (_S) phase. This is specific to glassy or amorphous states and herein could be attributed to a large difference in bond distances or disordering, for in particular _M phase.^[^ ^19^, ^22^ ^]^

### 2.4 Tracking the Reversible Electrochemistry of Na_1.2_Fe_1.2_PO_4_F_0.6__M

**Figure 6:**
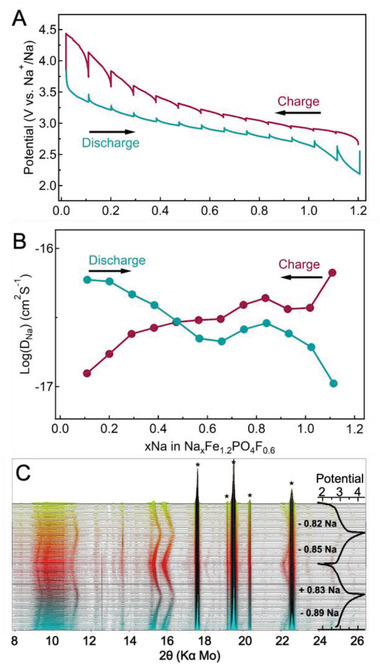
Na‐ion diffusion and phase reversibility during the charge and discharge process of Na_1.2_Fe_1.2_PO_4_F_0.6__M. A) GITT curves for the first cycle and B) diffusion coefficient of Na^+^ calculated from the GITT curves as a function of Na‐ion content. GITT was tested under a galvanostatic current pulse of C/80 for a duration of 6 h followed by relaxation at an open circuit for 24 h at each step. C) In situ XRD pattern evolution and corresponding potential profiles. Peaks of Al and Cu foil from the cell construct are marked by an asterisk.^[^ ^25^ ^]^

**Figure 7:**
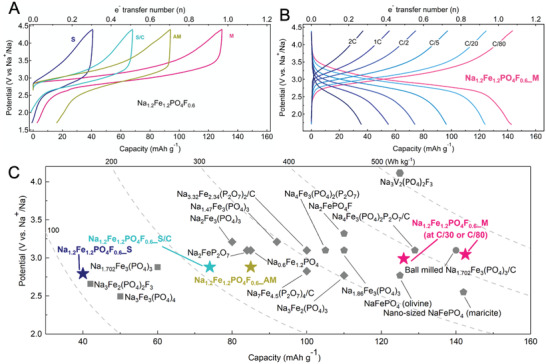
Comparative analysis of electrochemical performance. A) First cycle potential versus capacity galvanostatic plots for Na_1.2_Fe_1.2_PO_4_F_0.6__M phase and its stable (_S), carbon‐coated stable (_S/C), and amorphous (_AM) analogs. B) Rate performance of Na_1.2_Fe_1.2_PO_4_F_0.6__M electrode. C) Energy density comparison (performances considered at the material level) of various iron phosphate and iron fluorophosphate cathode materials for SIBs.^[^ ^9^, ^10^, ^24^ ^]^ The energy density guidelines can be considered as the product of average discharge redox potential as measured in a half‐cell configuration multiplied by the discharge capacity.

The charge and mass transfer kinetic behavior of Na_1.2_Fe_1.2_PO_4_F_0.6__M was examined using the galvanostatic intermittent titration technique (GITT, refer to Figure S5, Supporting Information, and associated text for analysis details). **Figure** **6**A depicts Na^+^ stoichiometry versus potential profiles for the charge and discharge process collected from GITT data. The profiles are similar to the galvanostatic charge–discharge data in **Figure** **7**, and the full Na^+^ content can be extracted and reinserted under these close‐to‐equilibrium conditions (charge or discharge at a current pulse of C/80 for 6 h followed by relaxation at an open circuit for 24 h at each step). Pronounced polarization occurs at end steps for both charge and discharge processes, indicating slower kinetics of Na^+^ diffusion when the full Na‐ion occupancy is attained. The diffusion coefficient of Na^+^ at each charge or discharge relaxation step was also calculated based on GITT data (Figure 6B). The coefficients are in the range of 10^−18^ to 10^−16^ cm^2^ s^−1^ within the potential window of 2.2–4.4 V (vs Na^+^/Na) and are comparable to values measured for other polyanionic compounds such as Na_0.9_FePO_4_ (8.63 × 10^−17^ cm^2^ s^−1^) and Na_3_V_2_(PO_4_)_3_‐C (NASICON, 2 × 10^−15^ cm^2^ s^−1^).^[^ ^23^ ^]^ Similar values for diffusion coefficients are obtained for Na_1.2_Fe_1.2_PO_4_F_0.6__S and _S/C, yet lower Na‐content exchange and higher polarization are monitored in the potential profiles (Figure S6, Supporting Information), which could be explained by the inactive Na^+^ sites in the crystal structure of the stable phase (Figure 5A).

To have further insight into Na^+^ extraction and insertion processes, in situ electrochemistry–XRD survey was performed.^[^ ^24^ ^]^ The main diffraction region and the corresponding potential profiles of the first two charge–discharge cycles at C/32 are shown in Figure 6C. Most of the diffraction peaks gradually shift to higher 2*θ* values when charging to 4.4 V (vs Na^+^/Na), and the opposite process occurs when discharging to 1.7 V, which can be explained by a solid–solution structural re‐arrangement and compaction during extraction, and vice versa during Na‐ion insertion. The good structural reversibility and stability demonstrate that the metastable structure of Na_1.2_Fe_1.2_PO_4_F_0.6__M is not remarkably impacted by the insertion and extraction of Na^+^ upon cycling, in good agreement with the cycling stability data (Figure S12A,D, Supporting Information).

Ex situ X‐ray photoelectron spectroscopy (XPS) finally confirmed the Fe‐centered redox of Na_1.2_Fe_1.2_PO_4_F_0.6__M (Figure S7, Supporting Information). In the Fe 2p spectra, 2p_3/2_ and 2p_1/2_ doublets located at around 710.7 and 724.5 eV are characteristic of Fe^2+^, while those at around 715 and 728.8 eV are of Fe^3+^. The doublets assigned to Fe^3+^ can be seen in the spectrum for the pristine sample, indicating surface oxidation, which is consistent with the fitting results of Mössbauer spectroscopy (Figure 5B and Table S3, Supporting Information). The area ratio of Fe^3+^ and Fe^2+^ peaks increases after charge and reversibly changes back to the original profile after discharge. The above observations evidence the reversible transition of Fe^2+^/Fe^3+^ redox couple upon Na^+^ extraction and insertion.

### 2.5 Comparative Electrochemistry Analysis of Na_1.2_Fe_1.2_PO_4_F_0.6__M, _S, S/C, and _AM

While the precise structural details of _M and _AM phases remain elusive at this stage, with the analyses of _M material mostly pointing to a mix of phases, the superior electrochemical material utilization is obvious (Figure 4B). We thus analyze and compare the Na^+^ storage performances of the perspective materials. In terms of the electrical conductivity of phosphate‐based polyanionic chemistries, here, the studied Na_1.2_Fe_1.2_PO_4_F_0.6_ chemistries make no exception and are intrinsically insulating, and as with other chemistries of this class, the carbon coatings can be applied to improve the charge transfer efficiency. Whereas the same logic could be pretended here, this has been revealed to be intrinsically impossible for the _M and _AM phases. Being metastable, further annealing resulted in the formation of the thermodynamically stable _S phase (Figure S8, Supporting Information). The carbon‐coated _S phase composite (Na_1.2_Fe_1.2_PO_4_F_0.6__S/C) in turn was prepared by adding a carbon precursor during the ball milling step (refer to Figure S9, Supporting Information, and associated content). The pore size distribution and specific surface areas of Na_1.2_Fe_1.2_PO_4_F_0.6__M, _S/C, and _S were characterized and compared using the Brunauer–Emmett–Teller technique (BET, Figure S10, Supporting Information) and gave the following values: 28, 17.9, and 4.9 m^2^ g^−1^, respectively. For the _S/C, growth of particle and aggregation is partially restricted, resulting in smaller particle size and a porous morphology, similar to _M material (Figure S11, Supporting Information).

The electrochemical performance of the respective materials was evaluated via similar galvanostatic charge–discharge protocols in Na half‐cells and the results are summarized in Figure 7 and Figure S12, Supporting Information. When tested at 5 mA g^−1^ (the equivalent of C/32), the Na_1.2_Fe_1.2_PO_4_F_0.6__S shows the lowest electrochemical active material utilization, with only 0.31 Na^+^ equivalents being possible to extract within the tested electrochemical window, applied cell construct and analysis procedure. The electrochemical activity is improved for the carbon‐coated Na_1.2_Fe_1.2_PO_4_F_0.6__S/C, with almost 0.56 Na^+^ being possible to reversibly extract and insert, most certainly originating from a better electrochemical charge transfer. Surprisingly, the metastable Na_1.2_Fe_1.2_PO_4_F_0.6__M, despite not being carbon coated, delivers the largest reversible capacity while also displaying the lowest polarization, with 0.98 Na^+^ equivalent being possible to reversibly exchange. One distinctive sloping plateau centered around 2.9 V (vs Na^+^/Na) can be seen in the potential profiles of both Na_1.2_Fe_1.2_PO_4_F_0.6__M and Na_1.2_Fe_1.2_PO_4_F_0.6__S/C. All display good cycling stability with a capacity retention of more than 90% after 50 cycles at a charge–discharge rate of C/32 (Figure S12A, Supporting Information). Although about 94 mAh g^−1^ (0.7 Na^+^ extraction) can be attained by the Na_1.2_Fe_1.2_PO_4_F_0.6__AM at the first charge, only 86% is reversible and it shows a gradual decrease in capacity upon cycling (Figure S12A,B, Supporting Information).

The rate capability measurements also revealed outstanding capacity reversibility and long‐term cycling stability of Na_1.2_Fe_1.2_PO_4_F_0.6__M despite not being carbon coated. At a low current density of 2 mA g^−1^ (the equivalent of C/80 rate), nearly the entire Na^+^ content (1.1 equivalents) can be reversibly extracted. The electrode maintains a high reversible capacity even at high rates, with 0.3 Na^+^ being possible to cycle at a rate of 2 C. Comparatively, the _S phase delivers a low capacity of also 0.3 Na^+^, yet when cycled at a slow rate of C/32. Whereas the higher capacity delivered by the carbon‐coated _S/C phase can be attributed to the improved charge transfer, the full material utilization in _M material is clearly not benefiting from this. Nanotexture and slightly higher surface area (_M > _S/C > _S) could be accounted for these improvements, given the possible intimate contact with carbon additive, as well as the smaller diffusion scale. However, these cannot fully support the observed improvements in _M and _AM materials. Since the metastable phases have been suggested to have larger molar volumes (or less compact) with higher symmetry crystalline structures, the hypothesis of favorable ion conduction for full Na^+^ utilization range is suggested here, supported by physico‐chemical analyses in previous sections. The long‐term cycling stability evaluated at a rate of C/2, following the variable‐rate test, further highlights the stability of this chemistry upon extended cycling and high current utilization.

Finally, the energy metrics of the best performing materials studied in this work are compared with analogous (Fe‐redox polyanionic) cathode materials in the literature, in terms of their average redox potential, reversible capacity, and energy density (all metrics considered at the material level) in Figure 7C.^[^ ^25^ ^]^ The Na_1.2_Fe_1.2_PO_4_F_0.6__M phase stands out amongst the best with an estimated energy density of 400 Wh kg^−1^ at the material level. Although this value remains inferior to those of vanadium‐based polyanionic cathode materials, iron presents the advantages of reduced costs and lower environmental burden.

## 3 Outlook and Significance of This Work

Metastable phases and materials for energy applications remain sporadically studied yet seem to hold significant promises.^[^ ^12a^ ^]^ The poor exploration comes from the fact that it is usually considered to work with stable thermodynamic phases as these are also much easier to prepare. It is also typically assumed that the thermodynamically stable phases may also be the best ones with the best electrochemical performances, as is indeed the case for most positive or negative battery electrode materials. However, many metastable (or transient) phases have been shown already to outperform their stable counterparts.^[^ ^12a^ ^]^ Furthermore essential is the richness of the multiple possibilities here, hence the multitude of the reaction channels a process can follow before reaching its lowest energy equilibrium might be endless (Figure 1B). Our work analyses only a few of these, available in the simple chemical, yet complex compositional and phases diagram of Na‐Fe‐PO_4_‐F. With a total of 14 materials presented in this work, out of which 6 are in the thermodynamically stable state, we find particularly enhanced electrochemical activity of the metastable ones.

The choice of Na_1.2_Fe_1.2_PO_4_F_0.6_ composition was guided by the capacity estimates, being the composition with equimolar Na and Fe content, with thus redox of Fe being possible to compensate by Na‐cation extraction, while also optimizing the Fe–PO_4_–F ratio to decrease the overall molecular weight. While the Fe to PO_4_ ratio was fixed in our work, it can be further presumed that additional compositions can be accessed by also altering this stoichiometry. Thus, the Na‐ and Fe‐rich (as compared to PO_4_ equivalents) Na_1.2_Fe_1.2_PO_4_F_0.6_ polyanionic framework was selected to attempt to understand the origins of its electrochemistry and structure–composition–property relationship. Within this same composition, we have accessed and analyzed as many as nine materials, all showing differences in characteristics, impacting their electrochemical properties significantly.

Although the exact structure for many of the presented materials remains elusive at this stage, with the hypothesis of the mixed‐phase composition being the most plausible one, in terms of energy density, the Na_1.2_Fe_1.2_PO_4_F_0.6__M clearly exhibits the best performance. With an energy content at the material level in the 400 Wh kg^−1^ range, it marks a leading position when compared to other reported iron phosphate‐based cathode materials for SIBs (Figure 7C). It is to be highlighted that for many of the reported materials, carbon coating, particle size controlling, and ball milling treatment techniques have been employed to improve the electrochemistry performances of otherwise poorly performing pristine materials (Figure 7C). The Na_1.2_Fe_1.2_PO_4_F_0.6__M is already at the forefront, being limited only by its moderate power performance given the absence of an electrically conducting film coating. This later will require ingenious approaches, as it should be based on low‐temperature methods so that the metastable material and activity are preserved. Although this particular metastable material is still not well understood at present, further in‐depth structural and electrochemical studies are required to unravel the complexity of the fundamental science for this promising cathode material, as is often the case for new materials.

## 4 Experimental Section

### Sample Preparation

The Na_0.6 +_ *_x_* Fe_1.2_PO_4_F*_x_* (*x* = 0, 0.2, 0.4, 0.6, 0.8, and 1) materials were synthesized via solid‐state reaction assisted by high‐energy ball milling. The stoichiometric amounts of Na_2_CO_3_ (≥99.5%, Sigma–Aldrich), NH_4_H_2_PO_4_ (99+%, Acros Organics), FeC_2_O_4_ 2H_2_O (99%, Sigma–Aldrich), and NaF (99%, Alfa Aesar), around 3.6–4 g in total, were first ball milled at 30 Hz for 20 min three times with 20 min pause under Ar atmosphere, using one stainless steel ball (25 mm diameter) in a 50 mL jar and high‐energy miller (Ball Mill BM500, Anton Paar). To obtain very fine powder precursors, the jars were opened and the caked precursors were scraped down using a spatula, followed by ball milling at 15 Hz for 10 min under an Ar atmosphere. For the carbon‐coated samples, 6 wt% (referring to the total amount of reactants) of stearic acid (extra pure, Acros Organics) was used as carbon source. The milled precursors were transferred into alumina crucibles and annealed at various temperatures and for different times under Ar flow (99.99% in purity) in a tube furnace with flange sealing system. The heating rate was 2 °C min^−1^, whereas the cooling process was natural. All samples were transferred into a glovebox (MBraun, <0.1 pmm O_2_ and H_2_O) with minimal exposure to air and stored therein for further analyses (for details refer to Figures S13 and S14, Supporting Information).

### Physico‐Chemical Characterization

Powder XRD (STOE DARMSTADT StadiP Transmission diffractometer system) using Mo K_*α*_ radiation (*λ* = 0.71073 Å) was employed to confirm the crystalline phases of the commercial starting materials, the ball milled precursors, and the synthesized samples. FullProf Suite software was used for PXRD indexing and refinement of Na_1.2_Fe_1.2_PO_4_F_0.6__S and FOX software for structure solution. Synchrotron powder XRD analysis was applied to Na_1.2_Fe_1.2_PO_4_F_0.6__M at the Material Science Beamline X04SA by a Measurement Service, PD Mesquik in the Paul Scherrer Institute.

TGA‐DSC‐MS was performed with N_2_ as carrier gas at a heating rate of 10 °C min^−1^ on a Mettler Toledo TGA/DSC 3+ STARe System, using alumina containers. ^57^Fe Mössbauer spectra were recorded at room temperature in transmission geometry mode with a constant acceleration mode conventional Wissel Mössbauer spectrometer equipped with a ^57^Co(Rh) radioactive source, a Reuter Stokes proportional counter detector, and a CMCA‐550 multichannel analyzer. All isomer shifts in Mössbauer spectra were given respective to *α*‐Fe. FT‐IR was carried out on pristine powders and spectra were recorded with a Shimadzu FTIR‐8400S spectrophotometer.

### Electrochemical Cell Assembly and Analysis

The working electrodes were fabricated by dry grinding the powders (70 wt%) with super P carbon (SP) as conductive agent (25 wt%) and poly tetra fluoroethylene (PTFE, powder, Sigma–Aldrich) as binder (5 wt%), followed by pressing a certain amount of the composite mix onto coin cell case (CR2032, SS‐316 steel). The typical mass loading was ≈6–9 mg cm^−2^. The electrochemical tests were performed in half‐cells (CR2032, SS316, coin cell format) with metallic Na as counter and pseudo‐reference electrode and one sheet of glassfiber (Whatman, GF/D) as the separator. NaClO_4_ dissolved in a 1:1 (v:v) mixture of ethylene carbonate (EC) and diethyl carbonate (DEC) containing 5 vol% fluoroethylene carbonate (FEC) was used as electrolyte.^[^ ^26^ ^]^ The assembly was carried out in an Ar‐filled glove box (MBraun, <0.1 pmm O_2_ and H_2_O). Galvanostatic charge–discharge tests were performed for the cells on Neware battery testing system at ambient temperature. GITT was carried out by charging or discharging at a current pulse of C/80 for 6 h followed by relaxation at an open circuit for 24 h at each step on BioLogic–Battery Cycling and Testing systems. In situ XRD patterns were acquired using an STOE Stadi P diffractometer operated in transmission mode, equipped with a Mo anode X‐ray source (details can be found in Figure S15, Supporting Information).

## Conflict of Interest

The authors declare no conflict of interest.

## Supporting information

Supporting Information

## Data Availability

The data that support the findings of this study are available from the corresponding author upon reasonable request.
