# Supplementary material for: New Cathode Materials in the Fe‐PO4‐F Chemical Space for High‐Performance Sodium‐Ion Storage
Source: Adv Sci (Weinh). 2022 May 26;9(22):2200924. doi: 10.1002/advs.202200924 (PMC9353465; doi:10.1002/advs.202200924)
Supplement: Supplementary file 1 — Supporting Information [file ADVS-9-2200924-s001.pdf]

Supporting Information

New Cathode Materials in the Fe-PO_4-F Chemical Space for High Performance Sodium-ion Storage

*Xuelian Liu,[§] Jiande Wang,[§] Mengyuan Du, Koen Robeyns, Yaroslav Filinchuk, Qi Zhu, Varun Kumar, Yann Garcia, Gheorghe Borodi, Cristian Morari, Jean-Francois Gohy and Alexandru Vlad**

§ Equal contribution

E-mail: alexandru.vlad@uclouvain.be

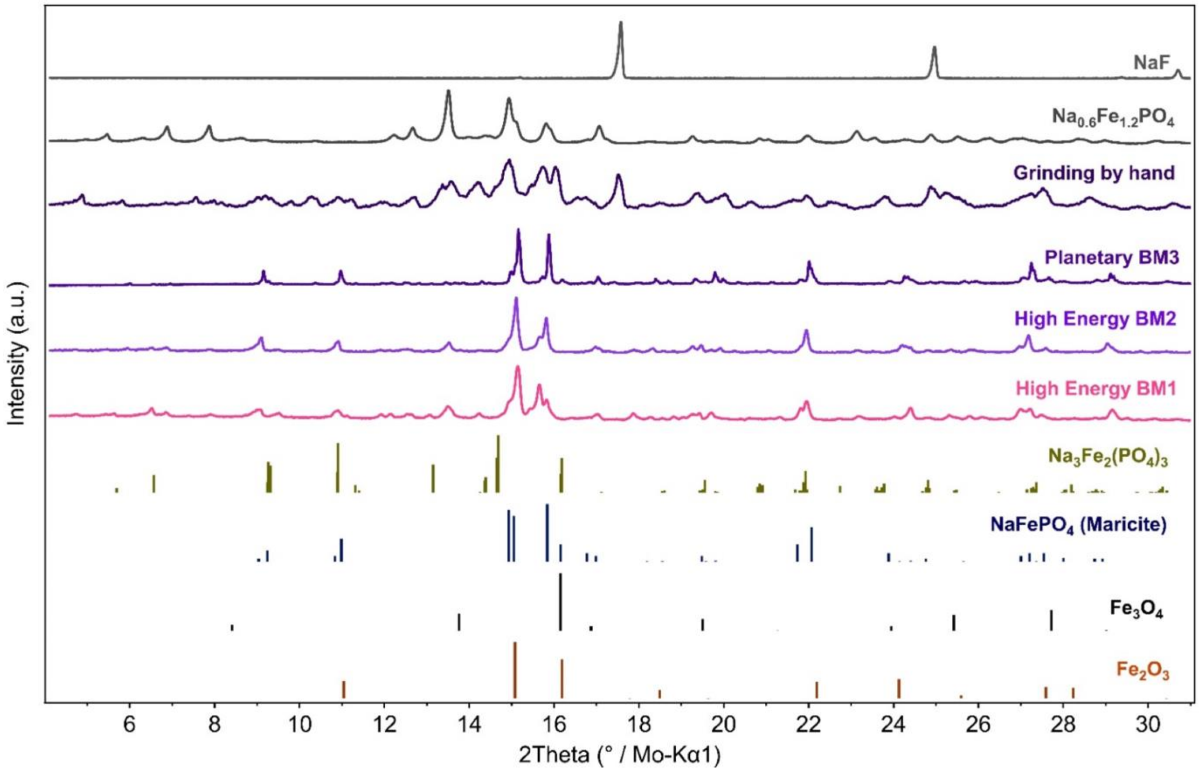

Figure S1. Comparison of PXRD patterns of $Na_{1.2}Fe_{1.2}PO_4F_{0.6}$ products using different approaches to prepare precursors and sintering under same conditions (550 °C for 12 h under Ar), together with PXRD patterns of NaF, stable phase of $Na_{0.6}Fe_{1.2}PO_4$ and the peak assignment of possible impurities.[1]

The two high-energy ball milled samples (BM1 and BM2) show similar patterns, whereas the pattern for planetary ball milled (BM3) is close to that of $Na_{1.2}Fe_{1.2}PO_4F_{0.6}$_S but with maricite $NaFePO_4$ and FeO_x impurities. Unreacted NaF can be observed in the pattern of product made by hand grinding. The rest of the peaks are broadened possibly requiring longer time or higher temperature for further reaction. This indicates that high-energy ball milling can induce effective mechanochemical activation for precursors and then accelerates the solid-state reaction during annealing. As a result, some new intermediate phases have been identified and are presented in this work.

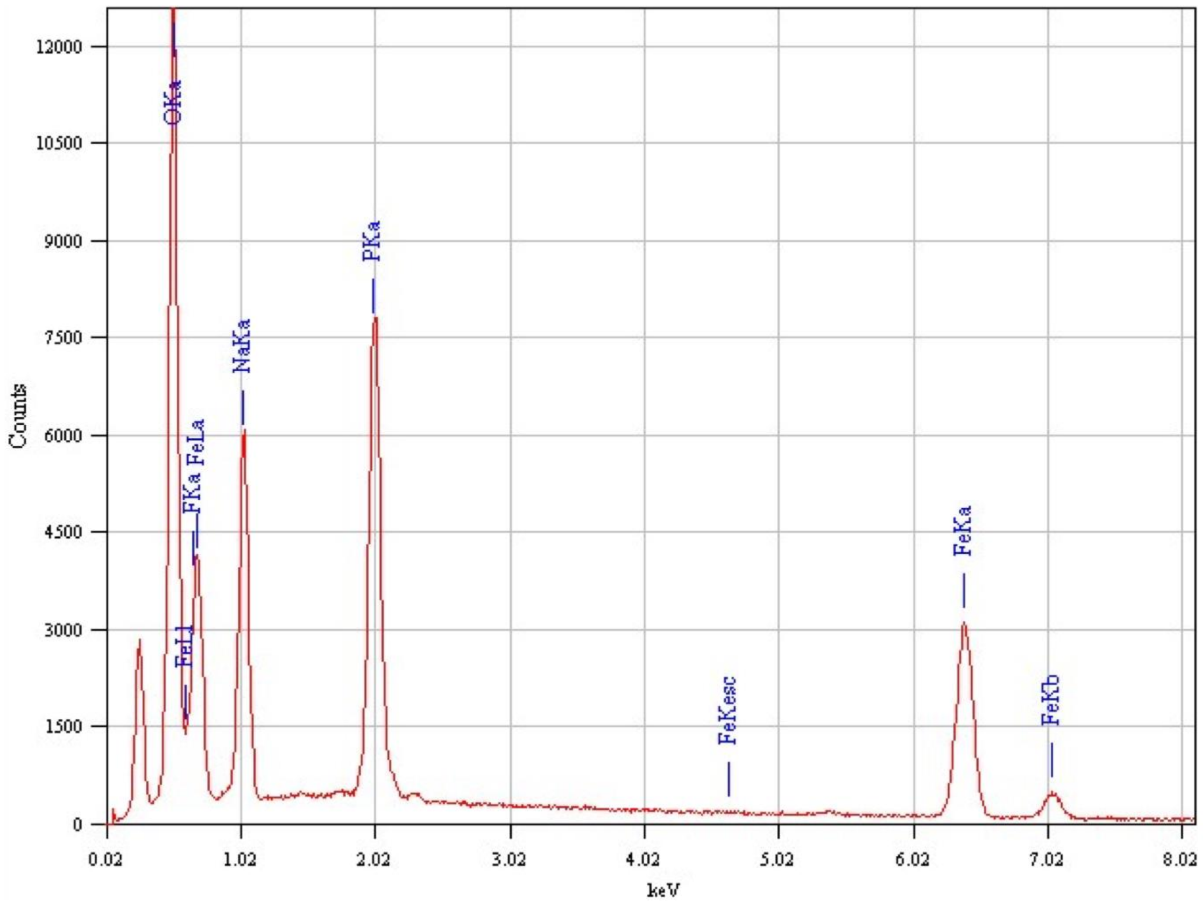

Figure S2. SEM-EDX of $Na_{1.2}Fe_{1.2}PO_4F_{0.6}$_M as an example for chemical composition analysis. Signals of all the elements in the compound can be detected.

Table S1. Elemental analysis of Na, Fe and P in $Na_{1.2}Fe_{1.2}PO_4F_{0.6}$_M measured by different techniques. The values from full-spectrum direct-reading inductively coupled plasma emission spectrometry (ICP, SPECTRO BLUE SOP) analysis are very close to theoretical composition (ratio of 1.2:1.2:1) based on the content of elements in starting precursor. The collected values from SEM-EDX are also close to the theoretical content.

Sample	Elemental ratio (Na:Fe:P)		
	Nominal	SEM-EDX	ICP
$Na_{1.2}Fe_{1.2}PO_4F_{0.6}$_M	1.2:1.2:1	1.38:1.47:1	1.13:1.18:1

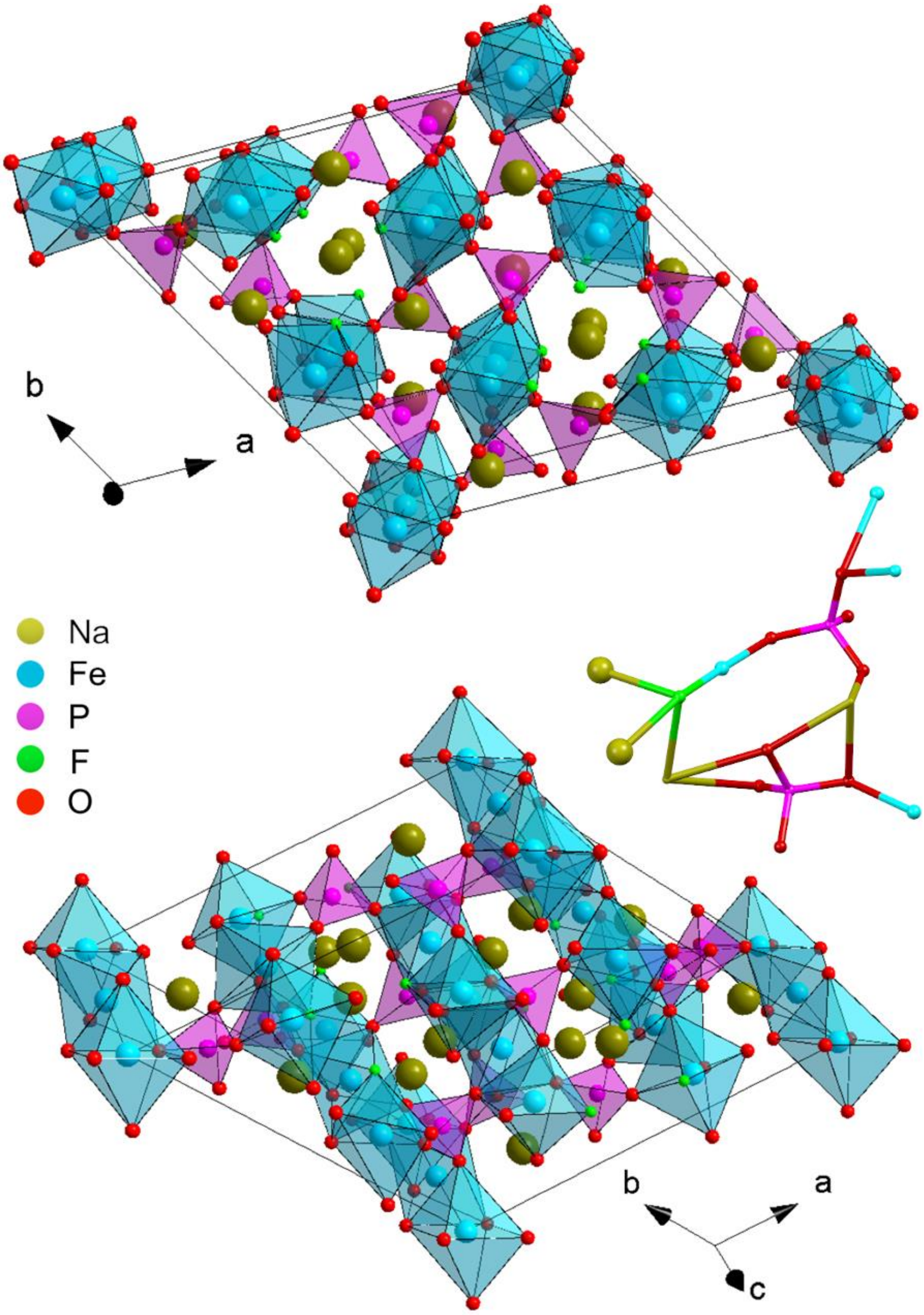

Figure S3. Crystal structure of $Na_{1.2}Fe_{1.2}PO_4F_{0.6}$_S from different view directions. The inset shows the asymmetric unit with isotropic temperature factors at the 50% probability level.

Table S2. Rietveld refinement results and atomic positions for $Na_{1.2}Fe_{1.2}PO_4F_{0.6}$_S.

**Phase: $Na_{15.16}Fe_{13.42}(PO_4)_{12}F_6$, Trigonal, space group P**						
**a = b = 13.62512 Å, c = 6.76738 Å, α = β = 90°, γ = 120°, V = 1088.007 Å^3**						
**Bragg R-factor = 7.76, Rf-factor** = 7.97						
**Atom**	**x**	**y**	**z**	**B**	**Occ**	**Mult**
Fe1	0.57625	0.70326	0.49169	1.206	1	6
Fe2	0	0	0	1.206	0.167	1
P1	0.62632	0.51077	0.75962	0.874	1	6
P2	0.81451	0.75076	0.25640	0.874	1	6
Fe3	0	0	0.5	0.874	0.070	1
Na2	0.33333	0.66667	1.01389	3.939	0.263	2
Na3	0.33333	0.66667	0.48632	3.939	0.263	2
Fe4	0.86854	0.57992	1.00128	1.206	1	6
Na5	0.06336	0.81959	0.23353	0.355	1	6
Na6	0.38990	0.49824	0.73160	0.495	1	6
F1	0.48203	0.71526	0.73737	0.955	1	6
O1	0.56052	0.44286	0.57589	0.955	1	6
O2	0.61573	0.61767	0.78139	0.955	1	6
O3	0.74996	0.54247	0.74447	0.955	1	6
O4	0.57221	0.43525	0.94477	0.955	1	6

O5	0.68678	0.70507	0.26484	0.955	1	6
O6	0.85249	0.71366	0.44402	0.955	1	6
O7	0.87839	0.88128	0.24794	0.955	1	6
O8	0.84638	0.70539	0.07313	0.955	1	6

CSD 2169355 contains the supplementary crystallographic data for this paper. These data can be obtained free of charge from FIZ Karlsruhe via www.ccdc.cam.ac.uk/structures.

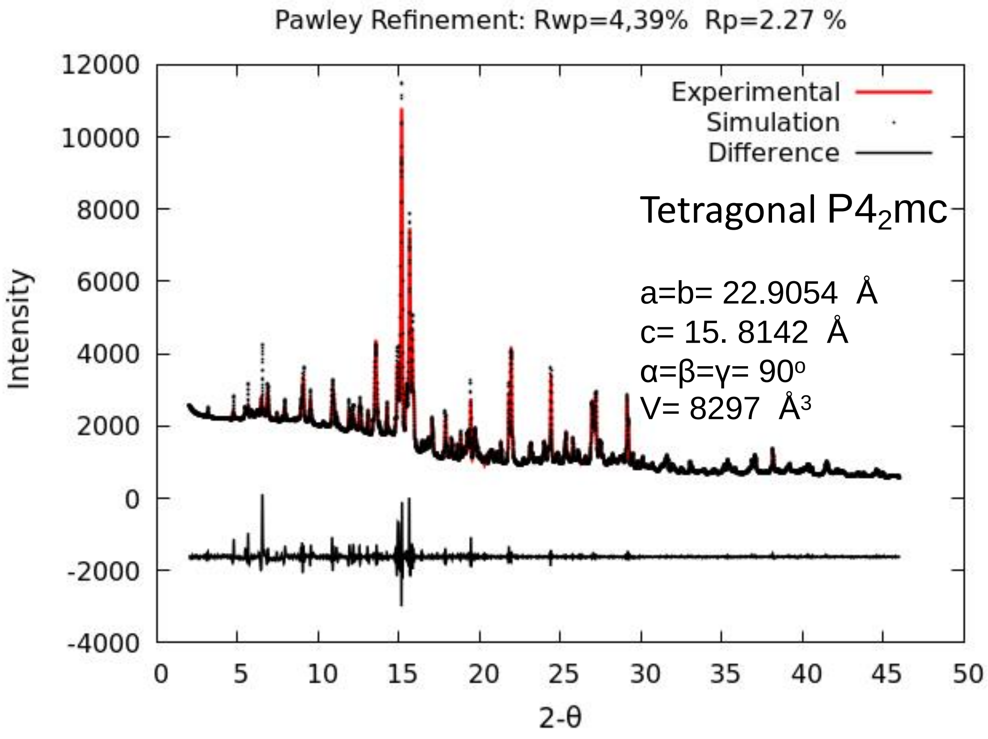

Figure S4. Synchrotron PXRD patterns and results of the Rietveld refinement of $Na_{1.2}Fe_{1.2}PO_4F_{0.6}$_M.

The powder diffraction pattern was recorded with Synchrotron radiation, wavelength being λ = 0.7085Å and the selected angular range 2θ between 2° and 46°. Various indexing programs such as Treor, Ito, Dicvol, X-Cell from Reflex modules, implemented in Materials Studio software were used. The solutions with the highest confidence factor were obtained with the X-cell computing program. We have obtained that the compound crystallizes in the tetragonal system with the lattice parameters a = b = 22.9054Å and c = 15. 8142Å, the unit cell volume V = 8297Å^3 and the space group $P4_2mc$. The first 48/49 diffraction peaks were indexed, even those with very low intensity (the number of diffraction lines that can be entered in the program as input data is maximum of 50). We have obtained: Rel. Fom = 0.82 and FOM = 304 as reliable factors. These reliability factors are acceptable considering that the diffraction peaks are very broad and the positioning of the diffraction maxima is more difficult. To confirm the correctness of the obtained space group, Pawley Refinement was applied. After Pawley refining, the Rwp = 4.39%, and Rp = 2.27% were obtained.

Table S3. ^{57}**Fe Mössbauer parameters obtained by fittings for $Na_{1.2}Fe_{1.2}PO_4F_{0.6}$_S, _M, _AM and $Na_{0.6}Fe_{1.2}PO_4$.** Isomer shift (δ) are given with respect to metallic α-Fe. DE_Q = quadrupole splitting. Γ/2 = half width of the lines. The parameter values for the $Na_{0.6}Fe_{1.2}PO_4$ are reproduced with permission from reference [2].

Sample	Site	δ (mm/s)	ΔE_Q (mm/s)	Γ/2 (mm/s)	Area fraction (%)
**$Na_{1.2}Fe_{1.2}PO_4F_{0.6}$_S**	**Fe(II)**	1.06	2.18	0.17	34
	**Fe(II)**	1.29	2.24	0.21	61
	**Fe(III)**	0.6	0.18	0.14	5
**$Na_{1.2}Fe_{1.2}PO_4F_{0.6}$_M**	**Fe(II)**	1.17	2.21	0.27	82
	**Fe(II)**	1.41	2.03	0.14	8
	**Fe(III)**	0.34	0.72	0.17	10
**$Na_{1.2}Fe_{1.2}PO_4F_{0.6}$_AM**	**Fe(II)**	**1.18**	**2.51**	**0.21**	40
	**Fe(II)**	**1.14**	**2.04**	**0.25**	45
	**Fe(III)**	0.51	0.40	0.20	15
**$Na_{0.6}Fe_{1.2}PO_4$**	**Fe(II)**	1.19	2.87	0.17	18
	**Fe(II)**	**1.18**	**2.45**	**0.23**	47
	**Fe(II)**	**1.12**	**1.94**	**0.25**	33
	**Fe(III)**	0.64	0.24	0.28	12

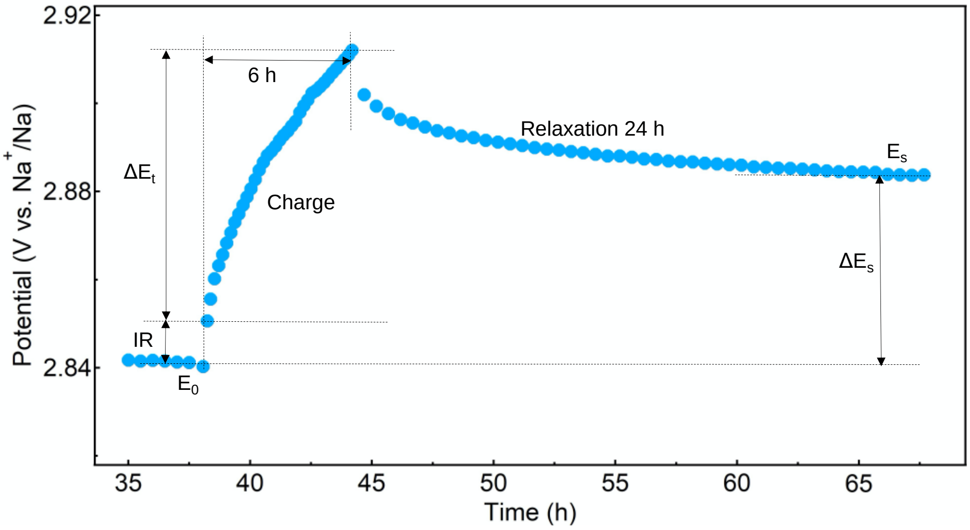

Figure S5. Schematic diagram of a single-step GITT experiment for $Na_{1.2}Fe_{1.2}PO_4F_{0.6}$_M at the current density of C/80 (2 mA g^{-1}).

Na^+ diffusion coefficients (D_{Na}) were determined for each step from GITT. The change in the steady-state voltage E_s is obtained by subtracting the original potential (E_0) from the steady-state potential (E_s). The process of the chemical diffusion is assumed to obey Fick's second law of diffusion. If sufficient small currents are applied for short time intervals, then $\mathrm{d}\boldsymbol{E}/\boldsymbol{d}\sqrt{\boldsymbol{t}}$ can be considered linear, as well as the coulometric titration curve over the composition range involved in that step. With these conditions, the equation of D_{Na} can be simplified to:[3]

$$D_{Na} = \frac{4}{\pi t}\left(\frac{n_m V_m}{S}\right)^2\left(\frac{\Delta E_s}{\Delta E_t}\right)^2$$

Here, t (s) is the duration of the current pulse; n_m (mol) is the number of moles; V_m ($cm^3\ mol^{-1}$) is the molar volume of the electrode; S (cm^2) is the contact area between the electrode and electrolyte; ΔE_s (V) is the steady-state potential change, due to the current pulse and ΔE_t (V) is the potential change during the constant current pulse, eliminating the IR drop. In this study, the V_m (65.5 cm^3 mol^{-1}) of the stable $Na_{1.2}Fe_{1.2}PO_4F_{0.6}$_S, calculated from its unit cell parameters and structure solution formula, was used as an approximation and the specific surface area (28.2 m^2 g^{-1}) and mass loading of the electrode were applied to estimate S for the D_{Na} calculation of $Na_{1.2}Fe_{1.2}PO_4F_{0.6}$_M.

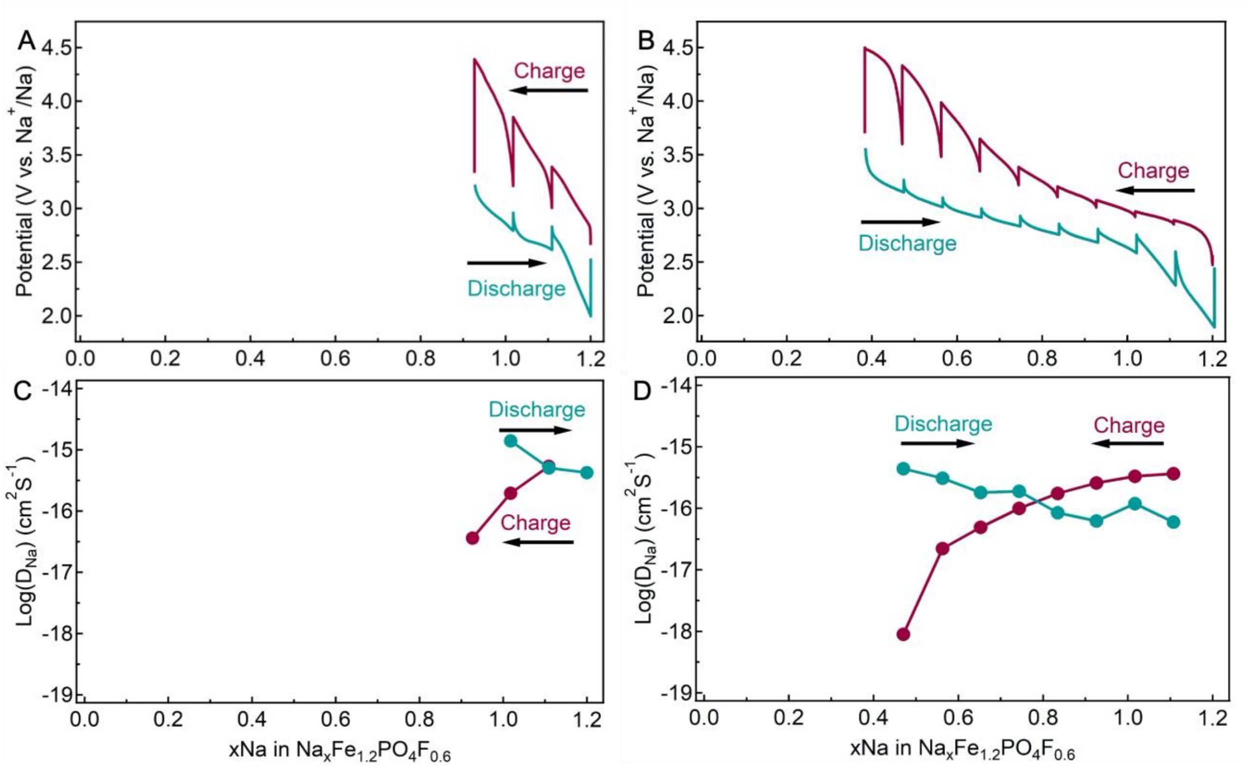

Figure S6. (A) and (B) GITT curves for the first cycle and (C) and (D) diffusion coefficient of Na^+ calculated from the GITT curves as a function of number of Na in the material for $Na_{1.2}Fe_{1.2}PO_4F_{0.6}$_S and _S/C, respectively.

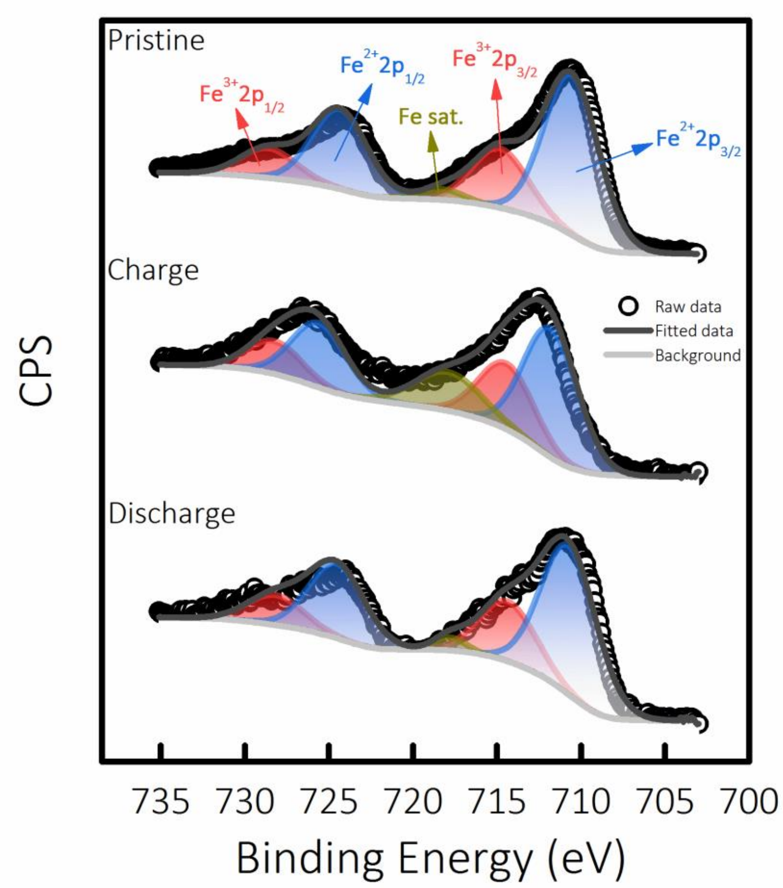

Figure S7. Ex situ x-ray photoelectron spectroscopy (XPS, ESCA) spectra of Fe 2p at charge and discharge states for $Na_{1.2}Fe_{1.2}PO_4F_{0.6}$_M, in comparison with the pristine sample.

For the ex situ XPS analysis, the cells were disassembled after charging or discharging, and the powders were collected, washed, dried and pasted onto holders in the glovebox, and the holders were sealed in plastic bag for transfer into analysis chamber. Then, XPS was performed for the powders on a SSX 100/206 spectrometer (Surface Science Instruments) using monochromatized and microfocused AlKα X-ray beam.

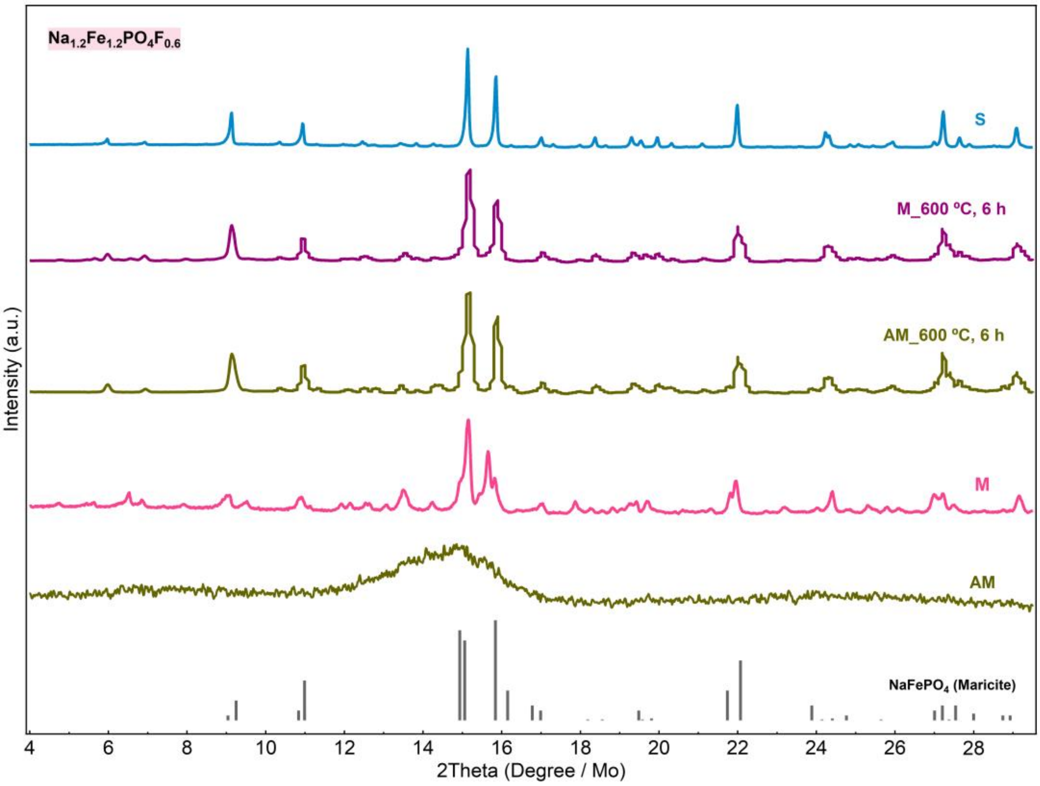

Figure S8. PXRD of $Na_{1.2}Fe_{1.2}PO_4F_{0.6}$_AM and _M annealed at 600 °C for 6 h, compared with the _AM, _M and _S counterparts.

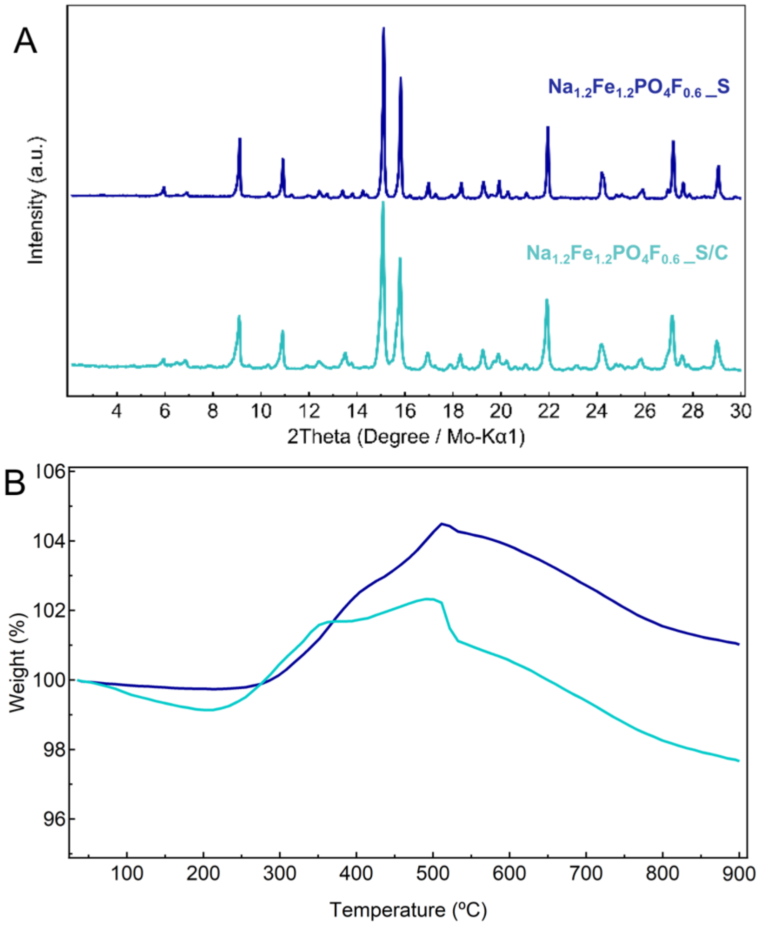

Figure S9. (A) PXRD and (B) TGA in air of $Na_{1.2}Fe_{1.2}PO_4F_{0.6}$ _S and _S/C. As expected, peak positions in the PXRD pattern does not change with a certain amount of carbon source added in starting materials before ball milling, but the peaks become broadened, resulting from its smaller particle size morphology. The carbon content of about 3 wt.% in the composite was confirmed by the TGA test.

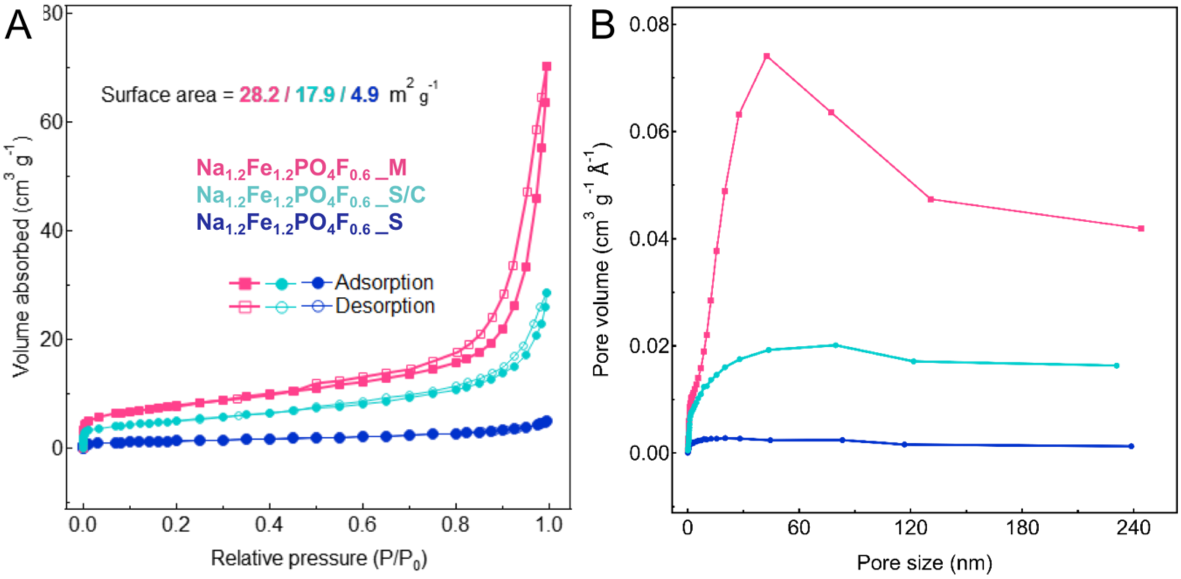

Figure S10. (A) Nitrogen-sorption isotherms (B) Pore size distribution of $Na_{1.2}Fe_{1.2}PO_4F_{0.6}$_M, _S and _S/C.

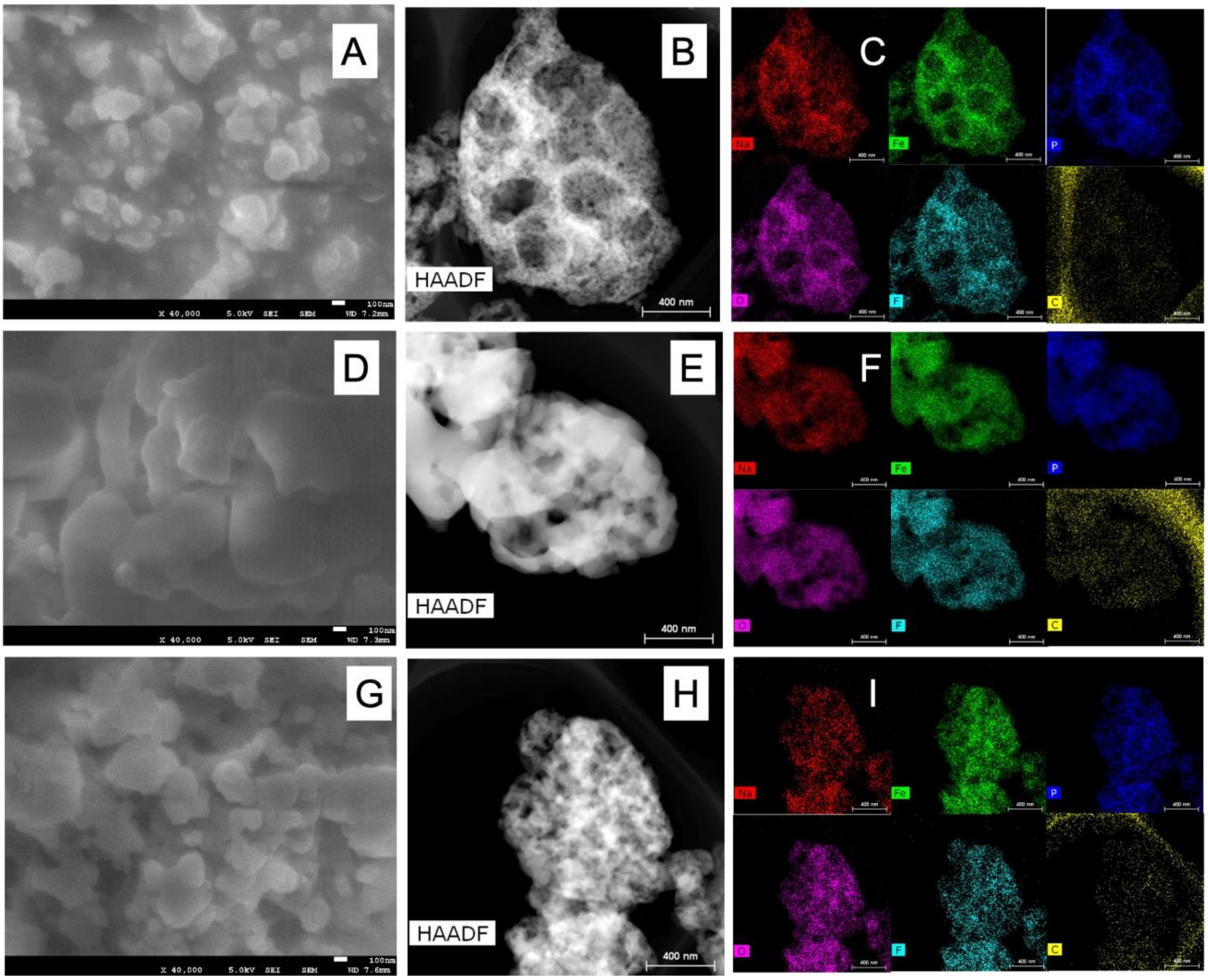

Figure S11. (A, D and G) SEM, (B, E and H) TEM-HAADF and (C, F and I) EDX mapping of elements images of $Na_{1.2}Fe_{1.2}PO_4F_{0.6}$_M, _S and _S/C, respectively.

The microstructure and morphology analyses were characterized by Scanning Electron Microscopy (SEM, JEOL JSM 7600F) and Energy dispersive X-ray spectroscopy (EDX, JEOL JSM 7600F), as well as Transmission Electron Microscope (TEM, Titan G2 60-300) and EDX (Titan G2 60-300) after dispersing the samples on holey carbon film on copper grids.

The small particle size of $Na_{1.2}Fe_{1.2}PO_4F_{0.6}$_M can be seen in the SEM image and it exhibits porous morphology with the pore size of ca. 100−200 nm, which is greatly noticeable in its HAADF image. It could be induced from the decomposition of the organic species releasing gases such as carbon dioxide, leading to abundant pore structures in the material. From the electrochemical point of view, the porosity structures are advantageous to penetration of the liquid electrolyte through the particles. With higher temperature or longer annealing time for synthesis, the $Na_{1.2}Fe_{1.2}PO_4F_{0.6}$_S

show dense particles in larger size. However, under the same annealing conditions, the particle growth has been inhibited by adding carbon source in starting materials before ball milling, resulting in its carbon coating counterpart ($Na_{1.2}Fe_{1.2}PO_4F_{0.6}$_S/C) with smaller and porous particle morphology. Those are accordant with the BET results in **Figure S10**. Additionally, the uniform distribution of Na, Fe, P, O, and F in typical particle for three compounds in the mapping images indicate that the raw reagents have been homogenously mixed in the ball milling process, ensuring the homogeneity of the obtained compounds.

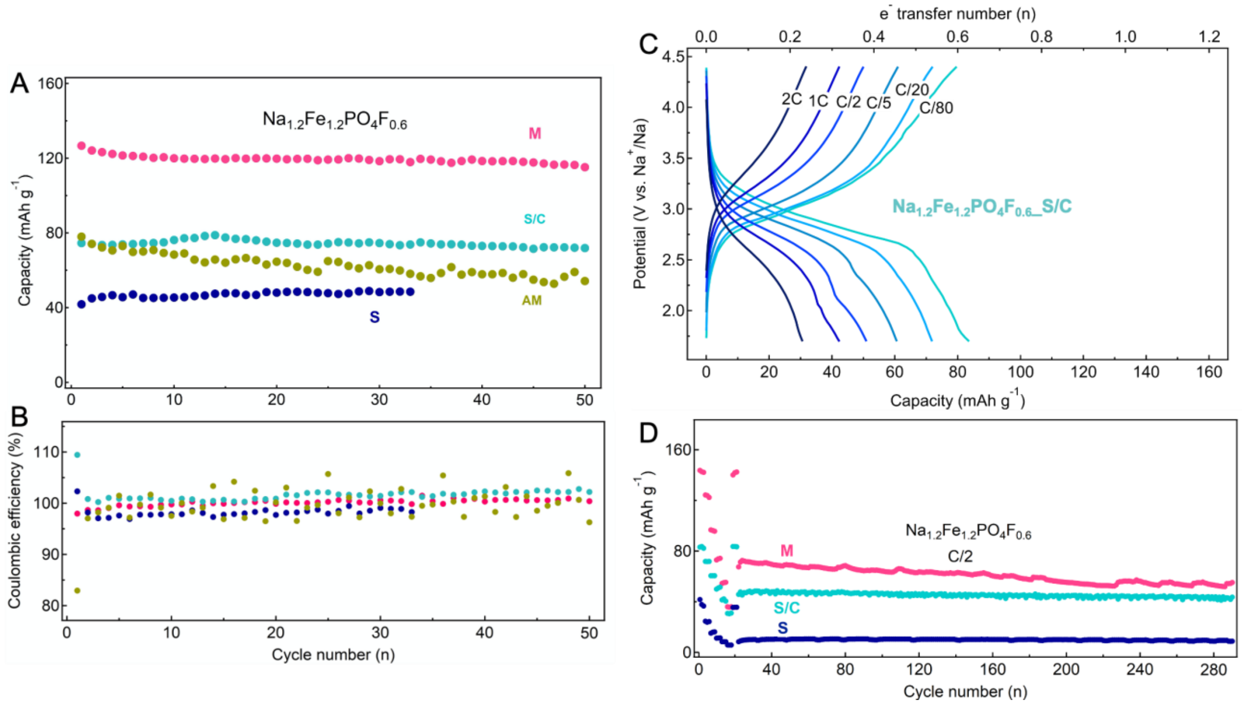

Figure S12. (A) Reversible discharge capacity and (B) coulombic efficiencies (CE) of $Na_{1.2}Fe_{1.2}PO_4F_{0.6}$_AM, _M, _S and _S/C cathode materials at 5 mA g^{-1} upon cycling. (C) Rate capability of the _S/C. (D) Reversible discharge capacity of $Na_{1.2}Fe_{1.2}PO_4F_{0.6}$_M, _S and _S/C cathode materials cycled at different current densities (C/80, C/20, C/5, C/2, 1C, 2C, and back to C/80, three cycles testing for each step), followed by long-term cycling stability at C/2 (1C = 160 mA g^{-1}).

Whereas the _AM shows a gradual decrease in capacity upon 50 cycles, the capacities for the other three materials stay stable. The CE for the _M, _S and _S/C cathodes are close to 100% over the period, except that the initial value for _S and _S/C are slightly higher than 100%, probably due to surface oxidation of materials obtained by annealing at high temperature or for very long time. In contrast, the CE for the _AM fluctuate severely around 100%. It suggests the unstable Na storage performance of this amorphous phase synthesised under low sintering temperature and the origins of this require further investigation.

With carbon coating, the _S/C cathode material delivers improved capacity and superior rate capacity. Its capacity is approximately half lower than that of _M at low current density of C/80, but the difference narrows as the current density grows. However, the electrochemical activity of this carbon coating stable phase is still limited with only 0.6 Na involved in the redox reaction, indicating that half of the Na in the material could be electrochemically inert in the crystal structure.

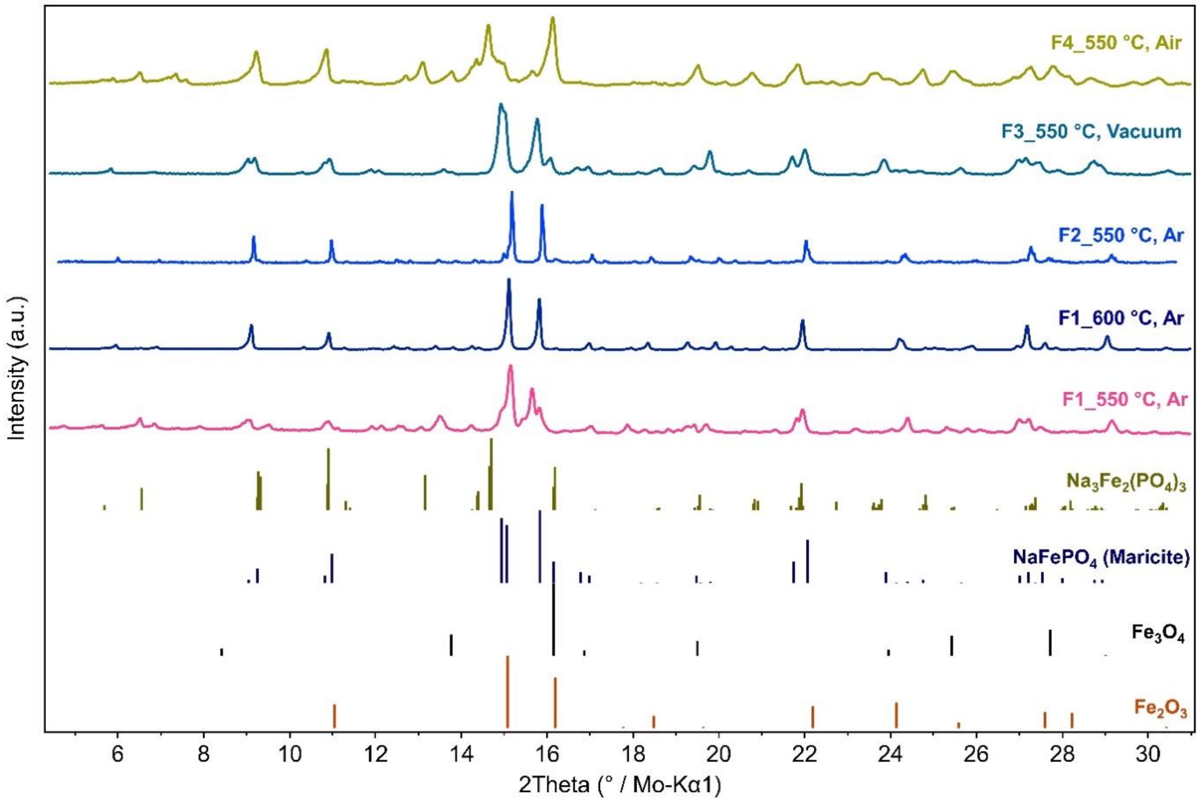

Figure S13. PXRD patterns of $Na_{1.2}Fe_{1.2}PO_4F_{0.6}$ products annealed for 12 h using different furnaces.

With the patterns compared, it can be seen that $Na_{1.2}Fe_{1.2}PO_4F_{0.6}$_M synthesized via Furnace 1 (F1) at 550 ^{o}C cannot be perfectly reproduced using other furnaces. It tends to form $Na_{1.2}Fe_{1.2}PO_4F_{0.6}$_S with $Na_3Fe_2(PO_4)_3$, maricite $NaFePO_4$ or FeO_x impurities. This could be because the decomposition of $FeC_2O_4·2H_2O$ varies in different furnaces or under different atmospheres.[4,5] To verify that, certain amount of $FeC_2O_4·2H_2O$ has been treated under the same condition as how the compounds were annealed in the furnaces, and PXRD patterns of the decomposition products are shown in **Figure S14**.

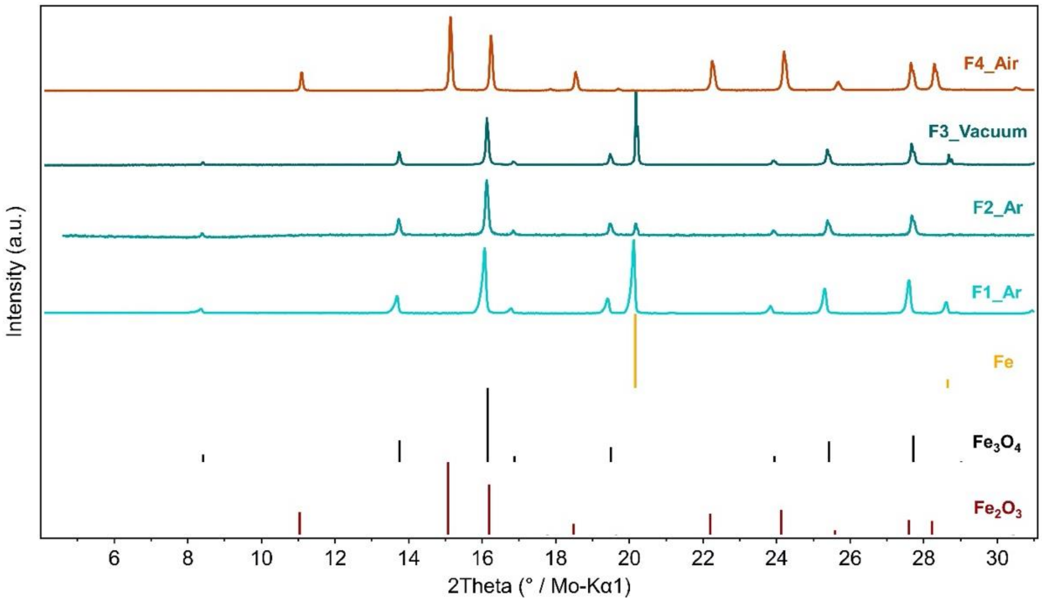

Figure S14. PXRD patterns of decomposition products of Fe(II)-oxalate using different furnaces.

It has been reported that its primary decomposition products are dependent on conditions and a mixture of Fe and Fe_3O_4 can form in an inert atmosphere ($FeC_2O_4 \rightarrow Fe + Fe_3O_4$).[4,6] The peaks of the products can be indexed to the mixture of Fe and Fe_3O_4 for those annealed under Ar or vacuum, but the intensity of Fe and Fe_3O_4 varies significantly for different furnaces. It should be noted that all the furnaces are tube furnace, but only F1 is equipped with flange for effective sealing. Rubber or ground glass stopper were used for homemade sealing system for F2 and vacuum system in F3 has also been tried. Without fast inert gas flow during annealing or when the vacuum is inefficient, the decomposition could be under reductive atmosphere due to the gas residue (CO) for the vacuum furnace. In contrast, if the sealing system is not effective enough and gas leaking would occur, the iron could be oxidized to higher valence by air, resulting in presence of residual Fe^{3+} phases and miss of desired phase. This could explain why the $Na_{1.2}Fe_{1.2}PO_4F_{0.6}$_M was failed to be prepared in other furnaces. The purity of the obtained compounds depends on the growth parameters, such as the temperature of calcination, and exposure time.[7] The is possible especially at high temperature for long time, resulting from a small amount of oxygen included in inert gas flow and/or residual air trapped in the small pores of the particles.

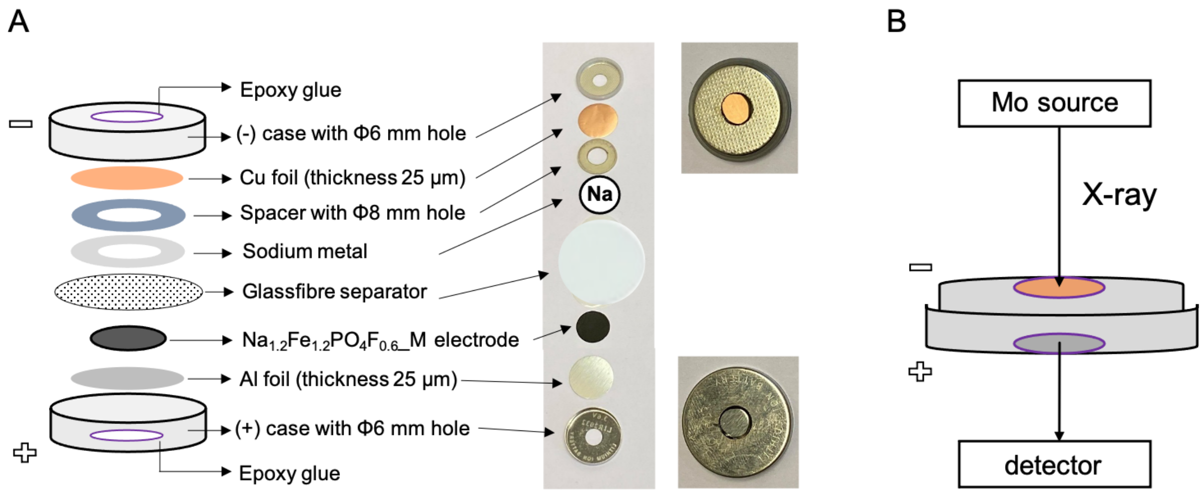

Figure S15. (A) Schematic representation and optical image of the in-house coin-cell used for in-situ XRD measurements. (B) Schematic illustration of XRD measurement according to transmission configuration used in this work (image reprinted with permission from reference).[7]

In situ PXRD patterns were recorded using a STOE Stadi P diffractometer operated in transmission mode, equipped with a Mo anode X-ray source (Kα radiation, operating at 50 kV – 40 mA) and Dectris Mythen 1K strip detector. Measurements were performed with a simple holder able to accommodate an in-house *in situ* coin cell. Firstly, one hole (6 mm diameter) is drilled in the center of both cases of the SS316 coin cell as well as the spacer (8 mm diameter hole). Al and Cu foils (thickness 25 μm) were fixed in the inner side of the cases with epoxy and used as X-ray partially permeable windows for positive and negative cases, respectively. The working electrode was prepared by mixing $Na_{1.2}Fe_{1.2}PO_4F_{0.6}$_M, Super P and PTFE with a composition ratio of 70:25:5 wt.% (5.3 mg in total). This composite was then pressed onto Al foil. Sodium metal disk was used as negative electrode, one sheet of glassfibre (Whatman, GF/D) as separator and $NaClO_4$ dissolved in a 1:1 (v:v) mixture of ethylene carbonate (EC) and diethyl carbonate (DEC) containing 5 vol% fluoroethylene carbonate (FEC) as electrolyte.

SI References

1. J. Lu, S.-i. Nishimura and A. Yamada, Electrochemistry Communications, 2017, 79, 51-54.
2. X. Liu, J. Wang, M. Du, Q. Zhu, K. Robeyns, X. Zhang, V. Kumar, J-F Gohy, Y. Garcia, A. Vlad, Batteries & Supercaps, 2022. (Online Version: https://doi.org/10.1002/batt.202100390)
3. Z. Shen, L. Cao, C. D. Rahn and C.-Y. Wang, Journal of The Electrochemical Society, 2013, 160, A1842-A1846.
4. D. Dollimore, Thermochimica Acta, 1987, 117, 331-363.
5. E. D. Macklen, Journal of Inorganic and Nuclear Chemistry, 1967, 29, 1229-1234.
6. V. Carles, P. Alphonse, P. Tailhades and A. Rousset, Thermochimica Acta, 1999, 334, 107-113.
7. D. Jugović and D. Uskoković, Journal of Power Sources, 2009, 190, 538-544.
8. L. Sieuw, A. E. Lakraychi, D. Rambabu, K. Robeyns, A. Jouhara, G. Borodi, C. Morari, P. Poizot and A. Vlad, Chemistry of Materials, 2020, 32, 9996-10006.
